# Simulating object recognition with the Standard Operating Procedures (SOP) model

**DOI:** 10.3758/s13423-026-02929-0

**Published:** 2026-06-04

**Authors:** Sergio N. Galarce, Edgar H. Vogel, Benjamin Keep, Jasper Robinson, Charlotte Bonardi

**Affiliations:** 1 Research Center on Cognitive Sciences, Faculty of Psychology, University of Talca, Talca, Chile; 2 School of Natural Sciences, College of Health and Science, University of Lincoln, Lincoln, UK; 3 School of Psychology, University of Nottingham, Nottingham, UK

**Keywords:** Animal and human associative learning, Recognition memory, Computational modeling

## Abstract

Object recognition is the ability to discriminate previously encountered objects from novel ones, typically inferred from exploratory preferences. It is usually treated as a form of recognition memory. A central idea is that both nonassociative and associative processes contribute, organized around priming: a stimulus is processed less, and therefore explored less, when its representation is already primed at the moment of encounter. Priming can arise from the stimulus’s own recent activation (self-priming) or from associative cueing by context or other stimuli (associative priming). Allan Wagner’s Standard Operating Procedures (SOP) model provides a quantitative framework for these two components, yet prior evaluations of this model have remained qualitative. Here, we specify a numerical implementation of SOP for the principal object-recognition procedures and their key manipulations (retention and interstimulus intervals, and spatial displacement). We then simulate three widely replicated experiments: spontaneous object recognition, relative recency, and object-in-place. The model quantitatively captures the canonical preferences (novel > familiar, remote > recent, displaced > nondisplaced) and aligns with the core features of recent datasets. Beyond fitting existing results, SOP has substantial heuristic value. Our analysis points to concrete future experiments that manipulate object locations, interstimulus spacing, distractor load, and arousal to dissociate associative from nonassociative influences.

Recognition memory in animals is variously described as the capacity to perceive a stimulus as familiar (Mandler, 1980), to judge whether it has occurred before (Warburton & Brown, 2015), and to distinguish it from a novel alternative (Nitka et al., 2020). In nonhuman animals, assessing this type of memory relies on the fact that, when faced with a choice between two objects, animals exhibit specific exploratory preferences. These preferences form the basis of the tasks used to evaluate object recognition.

One such preference is that rodents generally tend to explore novel objects over familiar ones (Berlyne, 1950). This behavior provides the ground for the spontaneous object recognition (SOR) task, where animals are more likely to choose an object they have not encountered before over one they have previously explored (Ennaceur & Delacour, 1988). Furthermore, when presented with two objects under similar conditions, animals often prefer the object presented earlier rather than the one offered more recently. This preference is assessed in the relative recency (RR) task (e.g., Mitchell & Laiacona, 1998). Finally, animals are more likely to explore a copy of an object located where that item has not been previously placed, compared with one located where it has already been placed. This behavior is evaluated using the object-in-place (OIP) task (e.g., Dix & Aggleton, 1999). Although there are more tasks for assessing recognition memory in nonhuman animals, we will focus on these three because they have been reproduced across many laboratories, they capture the core manipulations that make the model’s mechanisms transparent, and most variants can be mapped onto these procedures and interpreted using the same principles.

Despite the simplicity of these procedures and their effects, the underlying mechanisms of recognition memory are still controversial (Nitka et al., 2020). Some authors argue that recognition comprises two independent processes of *recollection* and *familiarity* (Aggleton & Brown, 2006; Mandler, 1980; Yonelinas, 2002). Recollection can be understood as the retrieval of contextual information about a past event, such as when, where, or how something happened, while familiarity is usually defined as the sense of knowing that one has seen something without recalling specific details about it (Yonelinas, 2002). Other researchers instead suggest that recollection and familiarity are a single process, and their dissociation only reflects differences in the magnitude of the memory trace relative to a particular decision criterion (Berry et al., 2012; Wixted, 2007). In what follows, we refer to these dual-process and single-process perspectives collectively as the classical view of recognition memory.

Alternatively, several authors (Honey & Good, 2000; Robinson & Bonardi, 2015; Sanderson & Bannerman, 2011) have explained recognition memory using the stimulus processing assumptions of the Standard Operating Procedures model (SOP; Mazur & Wagner, 1982; Wagner, 1981). In SOP, a stimulus comprises many representational elements. Upon presentation, some elements enter the active state (A1) and drive vigorous responding; they then decay into the passive state (A2), which encodes recency/expectancy and elicits weaker responding. On a later encounter, if any elements reside in A2, fewer elements are available to be activated into A1, and the net response is therefore weaker. If a proportion of stimulus elements are in A2 during stimulus presentation, this is referred to as stimulus priming.

Priming arises via two routes. Self-priming reflects lingering activation from the item’s own recent presentation. Associative priming reflects retrieval via long-term links to other cues, including context. These links develop when the to-be-associated representations are concurrently active via an associative contiguity rule that will be described in detail below. Thus, repeated presentations of an object in a given setting produce short-lived changes in responding through self-priming, whereas longer-lasting changes emerge as context–object associations that accumulate over time.

The relationship between SOP and recognition memory becomes evident when the target stimuli are treated as objects, the response is their exploration, and context is operationalized as specific locations in the arena. Figure 1 outlines these assumptions by showing, on the left, simplified depictions of the three experimental designs, and, on the right, descriptive outcome patterns intended to convey the relative contribution of associative and nonassociative priming across the three tasks.

**Fig. 1:**
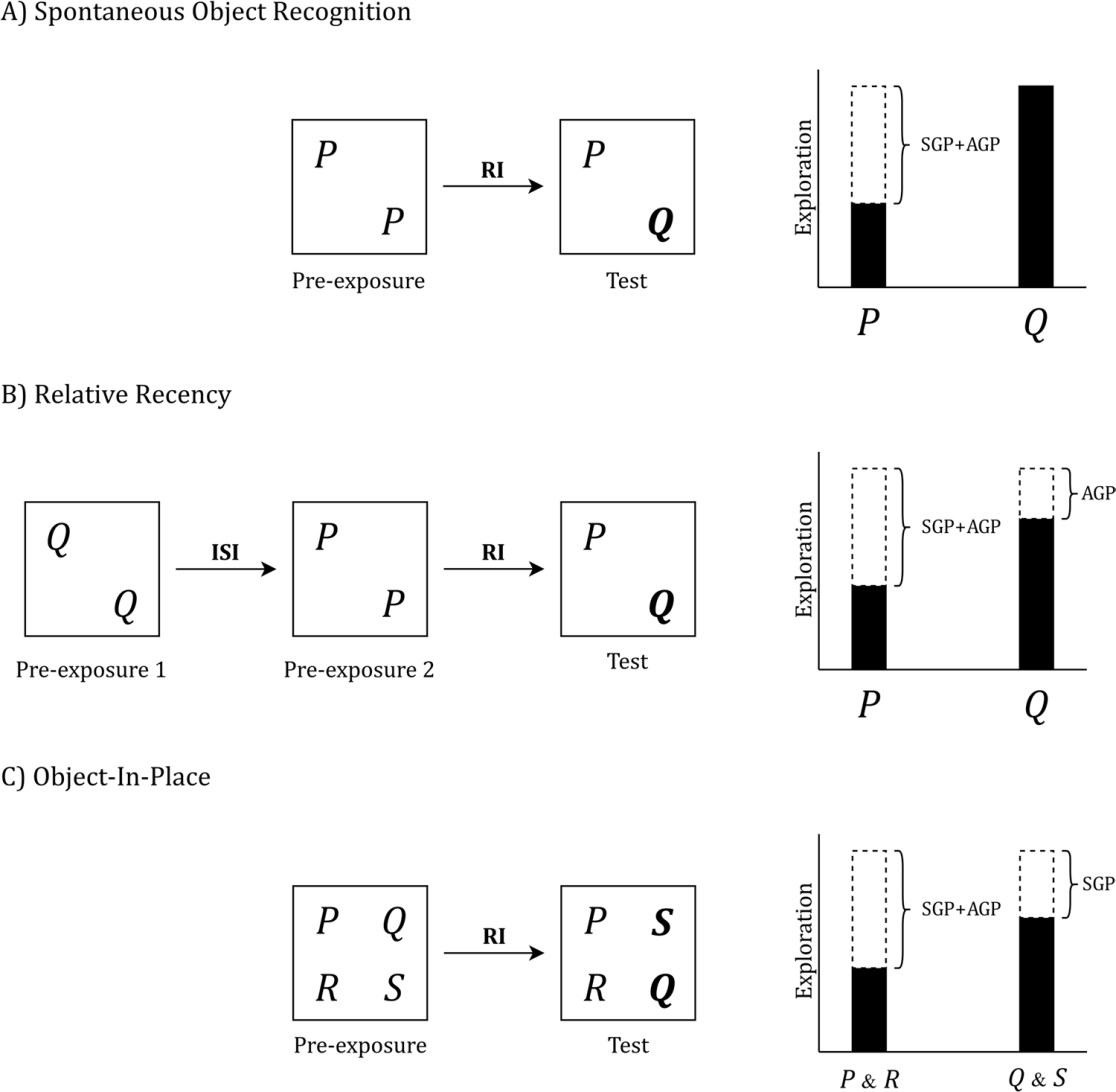
Prototypical designs of object recognition tasks and expected outcomes according to the SOP model. Each panel depicts an object-recognition task conducted in an arena, where copies of objects (P, Q, R, S) are placed at specific locations for free exploration. Left: simplified design of the procedure—**A)** SOR; **B)** RR; **C)** OIP. Boxes indicate object placements during preexposure and test; arrows indicate the interstimulus interval (ISI) within the sample phase and the retention interval RI before the test. Object labels in bold mark the items expected to receive greater exploration at the test. Right: expected test exploration and the SOP processes that generate it: SGP (self-generated priming from recent exposure) and AGP (associatively generated priming from context–object learning), where context is operationalized as location in the arena

Figure 1A illustrates the SOR task. In the sample phase, two copies of object P are placed in the arena for free exploration. After a retention interval (RI) in the same context, P is presented at test together with a novel object Q, and Q is typically preferentially explored. Within SOP, this preference arises because the familiar item P is primed in two ways: by its own recent encounter (self-priming) and by retrieval through its association with the context, here treated as location in the arena (associatively generated priming). In the standard SOR arrangement, both sources of priming apply only to P and are jointly determined by the same sample exposure and unchanged test context, so they covary and act in the same direction; without additional manipulations, such as changing the test context or varying the delay independently, their separate contributions cannot be identified.

Figure 1B illustrates the RR task. Two copies of Q are sampled first, then two copies of P. The test is the same as in SOR: P and Q are presented together in the same arena, and a reliable preference for Q is typically observed. In SOP this follows from the time course of self-priming, which is transient and declines with elapsed time since the last encounter. Because Q was sampled earlier, its passive-state priming has had more time to dissipate; at test it is primed less, more likely to reenter the active state, and therefore more explored. Because exposure duration and context are matched across samples, context–object associations should be comparable for P and Q; thus, in this schematic overview, the difference in exploration is attributed primarily to self-priming. However, SOP also allows an additional associative contribution when both samples occur in the same physical context. During the second sample, the context can retrieve the remote object (Q) into A2 even while it is absent, and pairing context A1 with object Q’s A2 accrues inhibitory learning, weakening the net context–Q association in an extinction-like manner. This would further reduce associative priming of Q at test and could therefore amplify the remote-over-recent preference (Robinson & Bonardi, 2015; Sanderson, 2016).

Figure 1C illustrates the OIP task. Four objects (P, Q, R, S) are placed at distinct locations in the arena, with location treated as context. After a RI, Objects P and R are returned to their original locations, while Q and S exchange positions. Because all items are sampled and tested together at the same delay, self-priming is effectively equal across objects. The critical difference comes from associative priming: P and R retain context–object associations with their original locations, which primes them at test and reduces the likelihood of their elements re-entering the active state. Q and S, being displaced, lack that associative support, are less primed, and more readily re-enter the active state, thus attracting greater exploration. This produces the characteristic preference for the displaced items Q and S.

The SOP model offers several advantages as a framework for understanding recognition memory. First, as a quantitative interpretation of a purely functional theory, the SOP model is applicable across species, including humans, making it a versatile tool for comparative research. Second, by situating recognition memory within a broader cognitive system, the model provides a unified explanation of memory and learning processes involved in various experimental procedures, including habituation and Pavlovian conditioning. Finally, the model generates quantitative predictions that can be experimentally tested (Bonardi et al., 2021).

In recent years, substantial research has focused on recognition memory within the framework of the SOP model (e.g., Bonardi et al., 2016; Nitka et al., 2020; Robinson & Bonardi, 2015; Sanderson, 2016; Tam et al., 2014, 2015; Whitt et al., 2012; Whitt & Robinson, 2013). However, these analyses have been primarily qualitative, which is not wholly satisfactory. SOP’s predictions depend on the relative strengths of two opponent processes so, for example, the parameters required to simulate one effect might be ineffective for simulating another. Demonstrating that the same instantiation of the model can simulate the full set of recognition phenomena while at the same time achieving its initial function of explaining associative learning is essential to confirm the viability of this account.

Therefore, the primary aim of this paper is to provide explicit quantitative simulations of SOP in the object-recognition domain using a computational implementation of the model. In addition, we include a quantitative assessment of model predictions that reports error metrics for model–data agreement and evaluates robustness to parameter variation via sensitivity analyses.

To further support researchers interested in exploring these predictions, we provide open-access code and a simplified set of instructions for implementing the SOP model. Given the challenges of quantitatively applying SOP, we believe that its impact has often been underestimated due to the difficulties associated with its precise numerical implementation. By making these resources available, we hope to facilitate further analysis and promote a more accurate assessment of the model’s theoretical contributions.

To achieve this, we formalize SOP as a system of coupled ordinary differential equations and state the assumptions that adapt it to object recognition. Objects are mapped to stimulus representations, locations in the arena serve as context, and exploration provides the response measure. We describe the activation, maintenance, and decay processes that generate quantitative predictions.

We then apply this formulation to the three canonical procedures: SOR, RR, and OIP. For each, we derive task-specific stimulus schedules and parameterizations, simulate representative published studies, and compare the model’s predicted exploration patterns with the reported outcomes. We complement these demonstrations with a quantitative assessment that evaluates prediction accuracy at both the object level and the contrast level. In addition, we provide a quantitative assessment of model predictions using error metrics and a separate set of global and local sensitivity analyses to evaluate robustness to parameter variation.

Finally, because we treat object recognition within a “habituation-like” framework, we outline how SOP extensions that have been useful for sensitization and dishabituation, including capacity limits under distractors and an arousal mechanism, could be adapted to object recognition. We present these as directions for future theoretical development and empirical work, rather than as formal evaluations in the present article.

## Overview of the object recognition literature: Species, stimuli, and timing

We reviewed studies using the three procedures described above. Rats are the most commonly used animals; two notable exceptions are mice (Sanderson & Bannerman, 2011) and humans (Gills et al., 2019; Mahoney et al., 2018; Nitka et al., 2020; Richmond & Nelson, 2009; Ryan & Cohen, 2004; Whitehead et al., 2018; Yeung et al., 2019; Zola et al., 2013). Stimuli varied widely, including “junk” objects such as cans, bottles, tins, glasses, and pots (Clark et al., 2000; Good et al., 2007; Nelson et al., 2011), construction blocks such as Duplo or Lego (Dix & Aggleton, 1999; Norman & Eacott, 2004, 2005), hardware items (Mitchell & Laiacona, 1998), cards (Whitt & Robinson, 2013), rubber ducks and baby bottles (Anderson et al., 2008), and virtual images presented on a computer screen (Nitka et al., 2020).

Most experiments were conducted in open-field arenas with objects placed at defined locations. Reported floor areas range from about 0.152 m^2^ (Nelson et al., 2011) to 1 m^2^ (Albasser et al., 2009; Dix & Aggleton, 1999). Maze-based variants also appear, including Y-mazes (Sanderson & Bannerman, 2011; Winter & Bussey, 2005) and radial mazes (Anderson et al., 2008; Chiba et al., 1997). In several maze studies, object exploration was replaced by exploration of familiar versus novel arms (Chiba et al., 1997; Sanderson & Bannerman, 2011). In the human study, recognition was assessed with eye tracking while participants viewed images on a screen (Nitka et al., 2020).

Typical sample phases range from about 30 s to 9 min. In several studies, the sample ended once a minimum exploration time was reached, usually 15–40 s (e.g., Langston & Wood, 2010; Norman & Eacott, 2004, 2005; Winters & Bussey, 2005). Tests most often last 3 min, with a range of 2–5 min; one study used a criterion of at least 30 s of exploration (Clark et al., 2000).

RI and interstimulus interval (ISI) vary widely, generally from ~10 s up to 24 h. Longer delays were rare: In their RR study, Mitchell and Laiacona (1998) used two 5-min sample phases separated by a 1-h interval and then tested retention after 1, 6, 24, 72, or 168 h. Reliable RR effects were obtained at 1–24 h but not at 72 or 168 h. By contrast, an SOR study employed a 3-week interval and still observed the expected pattern (Mumby, 2007). In the only human study (Nitka et al., 2020), events were very brief: they used 3-s samples and tests with ISIs/RIs of 0.5, 1, and 2 s in Experiment 1, and an ISI of 1 s with RIs of 1 and 10 s in Experiment 2.

In SOR, longer total sample exposure improves performance (Albasser et al., 2009). Performance here means stronger evidence of recognition, typically less exploration of the familiar item relative to the novel one. Spacing exposure across multiple brief samples rather than massing it in a single bout also enhances performance (Anderson et al., 2008; Sanderson & Bannerman, 2011; Whitt & Robinson, 2013). Increasing the RI reduces the novelty preference (Clark et al., 2000; Norman & Eacott, 2004, 2005; Tam et al., 2014), although effects have been observed even after 3 weeks (Mumby, 2007).

In RR, longer ISIs between the two sample phases improve performance (Hatakeyama et al., 2018; Tam et al., 2014). Evidence on the RI is mixed: Mitchell and Laiacona (1998) reported similar effects at 1, 6, and 24 h, whereas Hatakeyama et al. (2018) did not find reliable effects at 3, 15, or 75 min. Other studies reported recency effects with delays from 2 min to 3 h but did not compare different delays within the same experiment (Good et al., 2007; Hannesson et al., 2004; Nelson et al., 2011).

In OIP, the effect is robust with RIs from about 2 min to 2 h (Dix & Aggleton, 1999; Good et al., 2007; Langston & Wood, 2010). Only one study directly compared two RIs within OIP and reported no difference in performance (Tam et al., 2014).^1^

## Adapting SOP to object recognition

SOP was developed for Pavlovian conditioning and habituation, and its mathematical formalizations are well established (see Jorquera et al., 2024, for a full treatment). Here we adapt the framework to object recognition. We briefly restate the governing dynamics and specify the mapping from task components to model variables: objects are stimulus nodes, arena locations are context nodes, and exploration is the response measure. This mapping underlies the simulations that follow.

**Fig. 2:**
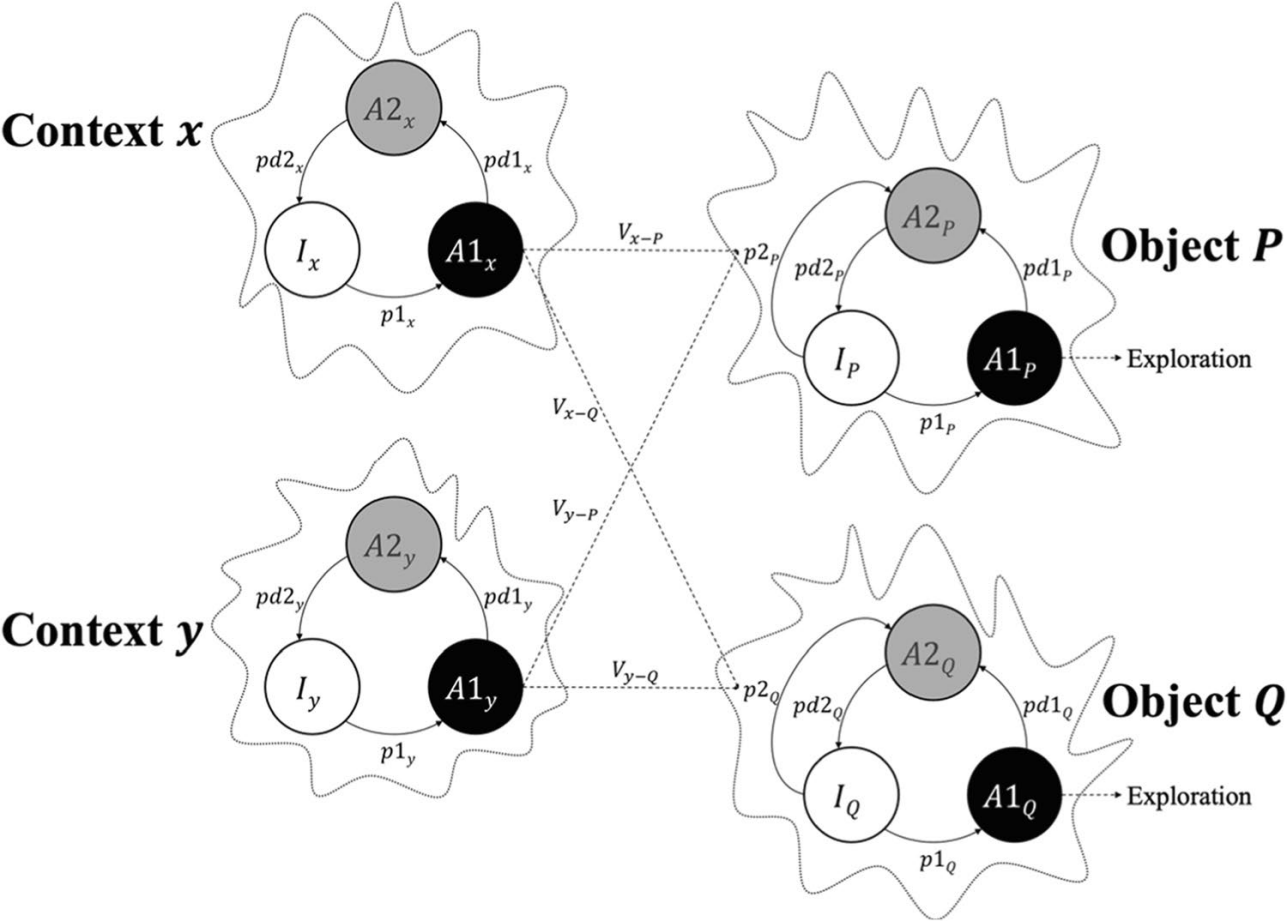
A visual representation of the SOP’s nodes in the tasks of object recognition. The objects P and Q and the contexts x and y are represented by nodes containing a set of theoretical elements that can be in one and only one of three states of activity: inactive, primary, and secondary (I, A1, and A2, respectively). Also, the transitional probabilities between the states of activity are depicted: p1 (function of the object’s intensity), pd1, and pd2 (object-independent decay functions). Through its associative link V, the contexts influence the activity of the objects by passing their elements directly from the I state to the A2 state with probability p2

Figure 2 illustrates this mapping with two object nodes (P, Q) and two context nodes (x, y). Each node contains a finite set of elements that can occupy exactly one of three states: inactive (I), primary activity (A1), or secondary (or refractory) activity (A2). State transitions depend on three probabilities: p1, which is driven by stimulus input, and pd1 and pd2, which reflect stimulus-independent decay processes. These dynamics yield two forms of priming: self-priming (SGP), which reflects residual activity after recent presentation of the same object, and associative priming (AGP), which reflects retrieval via learned links from contexts to objects (Vogel et al., 2019).

At any moment $t$, the proportion of elements of an event *i* (object or context) in each state can be represented as ${I}_{i}(t)$, ${A1}_{i}\left(t\right)$, and ${A2}_{i}(t)$, which always satisfies the following equality:

1 $${I}_{i}\left(t\right) \mathrm{+} {A1}_{i}\left(t\right) \mathrm{+} {A2}_{i}\left(t\right) \mathrm{=} 1,$$

with ${I}_{i}\left(0\right) \mathrm{=} 1$, and ${A1}_{i}\left(0\right) \mathrm{=} {A2}_{i}\left(0\right) \mathrm{=} 0$. When i is presented, a proportion of its elements move from I_i_ to A1_i_ with a probability of p1_i_, which is understood as a function of the intensity or salience of i. Once in the A1_i_ state, some elements move to A2_i_, with a probability of pd1_i_, and then back to I_i_, with a probability of pd2_i_. These parameters are independent of whether i is on or off and are interpreted as decay functions (Mazur & Wagner, 1982; Wagner, 1981). These assumptions allowed the SOP model to be mathematically formalized as a coupled system of ordinary differential equations (Jorquera et al., 2024; Uribe-Bahamonde et al., 2019), where the rates of change of the three states of activity are expressed as:

2 $$\frac{d{I}_{i}\left(t\right)}{dt} \mathrm{=} {pd2}_{i}(t){A2}_{i}(t)-{p1}_{i}(t){I}_{i}(t),$$

3 $$\frac{d{A1}_{i}\left(t\right)}{dt}\mathrm{=}{ p1}_{i}(t){I}_{i}(t)-{pd1}_{i}(t){A1}_{i}(t),$$

4 $$\frac{d{A2}_{i}\left(t\right)}{dt}\mathrm{=}{ pd1}_{i}(t){A1}_{i}(t){-pd2}_{i}(t){A2}_{i}(t).$$

Equations (1–4) are sufficient to capture SGP. Consider the node representing object P in Fig. 2. During object P’s presentation, the peak activity in A1_P_ is taken to be proportional to the total time spent exploring it. Following this initial presentation, a significant proportion of elements transition to the A2_P_ state, leaving fewer elements in the inactive state (I_P_) available to be promoted to A1_P_ during subsequent presentations. As a result, when P is presented again, the response, measured as exploration time, will be reduced compared with the first presentation. This effect diminishes as more time passes between presentations because elements gradually decay from the A2_P_ state back to I_P_. Consequently, the longer the RI, the less pronounced the reduction in exploration becomes.

Contextual cues can also generate secondary activity in an object’s representation through learned, unidirectional links from each context to each object. The net associative strength of a link is the difference between excitatory and inhibitory components that accrue simultaneously. Excitatory association grows in proportion to the co-occurrence of primary activity in the context and the object (A1_context_ with A1_object_). Inhibitory association grows in proportion to primary activity of the context together with secondary activity of the object (A1_context_ with A2_object_). For instance, for object P and context x, this is formalized by:

5 $$\frac{d{{V}^{+}}_{x-P}(t)}{dt}\mathrm{=}{{L}^{+}A1}_{x}\left(t\right){A1}_{P}(t),$$

6 $$\frac{d{{V}^{-}}_{x-P}(t)}{dt}\mathrm{=}{{L}^{-}A1}_{x}\left(t\right){A2}_{P}(t),$$

7 $${V}_{x-P}\left(t\right)\mathrm{=}{{V}^{+}}_{x-P}\left(t\right)-{{V}^{-}}_{x-P}(t)$$

Where V^+^_x-P_ and V^-^_x-P_ are the excitatory and inhibitory components, V_x-P_ is the net association from context X to object P, and L+ and L− are excitatory and inhibitory learning rate parameters, respectively. The same rules apply to links from other contexts and to links targeting object Q.

It is possible for more than one context to be associated with the same object. As shown in Fig. 2, context y also forms a link to P, denoted as V_y-P_. Let C be the set of contexts linked to P. Active contexts can promote elements directly from I_P_ to A2_P_ with a probability of p2_P_(t). We compute p2_P_(t), by aggregating the associative input from all linked contexts and truncating the result to the interval [0,1]:

8 $${p2}_{P}\left(t\right)\mathrm{=}min(1, \, max\left(0,\sum\limits_{c\in C}{A1}_{c}\left(t\right){V}_{c-P}\left(t\right)\right).$$

In Equation 8, A1_c_(t) is the primary activity of context c, and V_c-P_(t) is the net association from context c to object P. An inhibitory context (V_c-P_(t) < 0) does not by itself produce promotion to A2_p_(t). Its only effect is to offset concurrent excitatory inputs from other co-active contexts (those with A1_c’_(t) > 0 and V_c’-P_(t) > 0) by subtracting from the sum in Equation 8. Intuitively, the inhibitory context “prevents elements from entering A2_p_(t) by canceling part or all of the excitatory drive. If the aggregate input is ≤ 0, the truncation yields p2_P_(t) = 0 and no elements move from I_p_(t) to A2_p_(t); otherwise, inhibition simply reduces the proportion promoted by excitatory cues. Because p2_P_(t) is bounded [0, 1], inhibition cannot make p2 negative, only smaller.

Therefore, considering the effect of contextual cues, the activity of object P is expressed as follows:

9 $$\frac{d{I}_{P}\left(t\right)}{dt}\mathrm{=}{pd2}_{P}(t){A2}_{P}(t)-{(p1}_{P}(t)\mathrm{+}{p2}_{P}(t)){I}_{P}(t),$$

10 $$\frac{d{A1}_{P}\left(t\right)}{dt}\mathrm{=}{p1}_{P}(t){I}_{P}(t)-{pd1}_{P}(t){A1}_{P}(t),$$

11 $$\frac{d{A2}_{P}\left(t\right)}{dt}\mathrm{=}{pd1}_{P}{(t)A1}_{P}(t)\mathrm{+}{p2}_{P}(t){I}_{P}(t){-pd2}_{P}(t){A2}_{P}(t).$$

These equations can explain the effects of the AGP mechanism. Exposure to the object P in either context x or context y will cause the latter to be associated with the former. Moreover, because these context–object associations drive a larger proportion of elements out of the inactive state I_P_ and into the refractory state A2_P_, there are fewer elements available to enter the primary activate state A1_P_, which decreases its response and therefore reduces exploration of P.

Four other points need to be considered concerning Fig. 2. First, all the above also apply to object Q. Second, the association between a context and an object will only occur if the object and context have nonzero A1 or A2 activity. In other words, the links shown in the figure should be interpreted as potential associations, rather than established associations.^2^ Third, links in the model are defined as unidirectional. This does not preclude reciprocal influence: a bidirectional relation would be represented as two distinct links (e.g., V_x-P_ and V_P-x_), whose magnitudes need not be correlated because they are computed separately. Accordingly, in this simplified formalization, p2 is defined for object nodes but set to zero for context nodes, because context nodes are assumed not to receive associative input from objects or from any other source. For that reason, no p2 arrow is shown for context x or y, so no arrow is shown for context x or y. In principle, context x and context y can also be activated through associative links with other stimuli (including, but not limited to, P and Q), and objects could, in turn, influence contexts through separate links. However, we omit these possibilities here to keep the exposition focused on context→object links, in line with standard Pavlovian practice: analyses concern the influence of the CS (our contexts) on the US (our objects), not the reverse. Fourth, for the same reason, we do not include object-to-object associations in the present article. That is, objects are treated as unconditioned stimuli that determine exploration via their own activation dynamics and their links with contexts, but they do not serve as cues that form associations with other objects. Object-to-object associations may nonetheless be important for explaining some findings in RR and temporal order procedures, and we return to this possibility in the Discussion (see Sanderson, 2016).

In summary, object P and object Q can be considered as unconditioned stimuli whose activation probability, and therefore degree of exploration, depends on both their own presentations and their potential associations with context x and context y, which can be understood as conditioned stimuli.

## Simulations

We simulated conceptual and empirical versions of the three procedures in Python. All event times (stimulus durations, ISIs, sample/test lengths) were specified in real time (seconds/minutes). We integrated the model’s differential equations using the fourth-order Runge–Kutta method with a fixed time step of dt = 3 s.

Choosing dt = 3 s balances temporal resolution against computational cost and visualization limits: if dt is too large, second-scale transients are underresolved and become indistinguishable in long-duration simulations; if dt is too small, the number of integration steps grows linearly with duration (steps = duration/dt), which substantially increases runtime without materially changing the outputs. Given that in actual experiments the sample and test phases typically last 5 and 3 min, respectively, and effects unfold over minutes to a few hours, dt = 3 s provides adequate resolution while keeping simulations tractable (1 min = 20 steps, 3 min = 60, 5 min = 100, 1 h = 1,200). We validated dt = 3 s by comparing RK4 trajectories with analytical (exact) solutions and observed negligible error at this step (Galarce et al., 2026).

As a first approach, instead of conducting any sort of parameter fitting or optimization, we adopted a straightforward strategy mostly following Vogel et al. (2019) and Jorquera et al. (2024) by setting p1_OBJECT_ = 0.75, p1_CONTEXT_ = 0.25, pd1_OBJECT_ = pd1_CONTEXT_ = 0.1, pd2_OBJECT_ = pd2_CONTEXT_ = 0.02, L^+^ = 0. 25, and L− = 0.025. Three key aspects of this decision might be worth highlighting. First, we set the salience of objects (p1_OBJECT_) at a higher value than that of contexts (p1_CONTEXT_), assuming that the context is a behaviorally more neutral stimulus. Second, the assumption that pd2 is smaller than pd1 for both the objects and the context guarantees that A2 activity lasts longer than A1 activity, a key factor for all priming effects (Wagner, 1981).

Lastly, we set L+ = 0. 25 and maintained a 10:1 ratio (L+ > L−). This proportion promotes net excitatory context–object associations and aligns with associative phenomena beyond object recognition (Vogel et al., 2020). The absolute L+ value is deliberately larger than in prior SOP simulations because object-recognition procedures demand rapid learning from a single sample/trial, unlike domains such as habituation (Uribe-Bahamonde et al., 2019) or conditioning (Vogel et al., 2019), where learning accrues gradually across many trials.

Our aims in this section are twofold: (1) to illustrate how SOP operates when applied to object recognition and (2) to assess how well it captures patterns reported in empirical studies. We first present conceptual simulations with simplified schedules to isolate the roles of A1, A2, self-priming, and associative priming in SOR, RR, and OIP. These demonstrations are not tied to any single experiment; instead, they are intended as a didactic depiction of how SOP adapts to the canonical manipulations used in these tasks, without including incidental experimental details.

We then simulate four representative studies (Good et al., 2007; Sanderson & Bannerman, 2011; Tam et al., 2014; Whitt & Robinson, 2013), reproducing their sampling schedules, ISIs, RIs, and test windows as closely as our time calibration permits. The dependent variable, exploratory behavior, is represented by peak A1 activity, which we treat as proportional to exploration in the model. Model parameters (p1, pd1, pd2, L+, L−) are held constant across studies (no fitting), providing a direct check on whether a single specification accounts for the reported patterns.

All simulations were deterministic, meaning that repeated runs of the same simulation produced identical results. Thus, the simulations did not incorporate stochastic variability, subject-level variability, or error variance; instead, they reflected the model’s expected values. In cases where simulations were used to reproduce empirical procedures, results were averaged across counterbalanced order permutations to avoid order effects. Any variability across these permutations would reflect only order-related differences in model output, not sampling error or individual variability. Because such variability was negligible and did not affect interpretation, it is not shown. The reported simulations should therefore be understood as expected model outputs, suitable for comparison with the central tendencies observed in the empirical data.

## Conceptual simulations of SOP in SOR, RR, and OIP

**Fig. 3:**
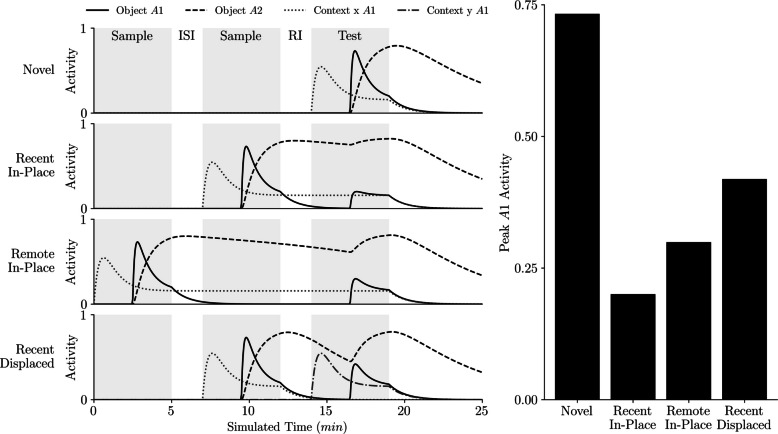
Demonstration of SOP processes across object recognition conditions. A single timeline is used for all rows: sample, ISI, sample, RI, test. ISI is the interstimulus interval between the two samples, and RI is the retention interval between the second sample and the test. Sample and test are 5 min each; ISI is 2 min; RI is 2 min. In each row, the left side plots SOP traces for the tested object A1 (primary) and A2 (secondary), together with the contexts’ A1 states, and the right side shows a bar summarizing predicted exploration as the object’s peak A1 at test. Row 1 (novel) shows an object not sampled until the test. Row 2 (recent in-place) shows an object sampled recently and tested in the same context in which it was sampled. Row 3 (remote in-place) shows an object sampled earlier in time and tested in the same context in which it was sampled. Row 4 (recent displaced) shows an object sampled recently and tested in a different context from the one in which it was sampled. Task mappings: SOR compares novel and recent in-place; RR compares recent in-place and remote in-place; OIP compares recent in-place and recent displaced

Figure 3 offers a single, integrated demonstration of how SOP dynamics give rise to typical object recognition outcomes in the SOR, RR, and OIP tasks. All conditions follow the same schedule: sample, ISI, sample, RI, test. This alignment allows differences in predicted exploration to be interpreted under common processes and timings. In each row, the left panel plots the evolving A1 (primary activation) and A2 (secondary activation) for the tested object, together with the contexts’ A1 states. These trajectories make two mechanisms explicit: (i) self-priming, where carryover in A2 suppresses a stimulus’s subsequent A1 at test; and (ii) associatively generated priming, where context to object links formed during sampling elevate the object’s secondary activation before the test, provided the test occurs in a context where such an association was formed. The bar on the right collapses these dynamics into the quantity we use to predict exploration: the object’s peak A1 during the test epoch.

The four rows instantiate the canonical task comparisons within this unified frame. In the *novel* condition, the tested object has not been sampled, so A2 carryover is absent and no supportive context to object links exist; the test therefore evokes a large A1, predicting high exploration. In the recent in-place condition, the object was sampled very recently and is tested in the same context in which it was sampled, so it is doubly primed; A2 carryover is high and the original context activates the object’s elements into A2 via learned links, yielding the smallest A1 and the lowest exploration. The remote in-place condition is also tested in the original location, preserving contextual activation, but the longer delay since sampling allows more A2 to dissipate, producing a larger A1 than in the recent condition. Finally, the recent displaced condition keeps recency constant while removing contextual support; without the original place, associative priming is reduced and A1 rises relative to the recent in-place condition.

These contrasts map directly onto standard behavioral tasks. SOR compares novel versus recent in-place (novel > familiar), isolating the difference between minimal and strong priming. RR compares recent in-place versus remote in-place, holding place constant to emphasize differences in self-priming due to time since sampling (remote > recent). OIP compares recent in-place versus recent displaced, holding recency constant to reveal the role of contextual associations (displaced > in place).

## Simulating multiple tasks from Good et al. (2007)

We focus on Good et al. (2007) because it examines object recognition across the three core tasks (SOR, RR, OIP) and also introduces a combined variant that manipulates both temporal and spatial order, termed RR enhanced by object in place (RR + OIP). This breadth allows us to test the SOP account within a single experimental framework and to compare predictions across closely related procedures.

RR + OIP includes two preexposure samples. In the first sample, objects P and Q are placed at two arena locations (e.g., the left and right rear corners). In the second sample, objects R and S occupy the two remaining locations (e.g., the left and right front corners). At test, all four objects are presented simultaneously; Q and S are displaced by exchanging locations, whereas P and R remain in their original positions. The expected preference is that Q attracts the most exploration because it was encountered earlier and is displaced at test, while R attracts the least exploration because it was encountered more recently and is not displaced.

The study used a within-subjects design in which each animal experienced SOR, RR, OIP, and RR + OIP in sequence. Good et al. (2007) compared sham-operated rats with rats bearing excitotoxic hippocampal lesions; here we simulate the sham-operated group only. Before the recognition tasks, each rat received two 10-minute habituation sessions to acclimate to the arena. For SOR and OIP, the preexposure phase consisted of a single 5-min sample. For RR and RR + OIP, there were two 5-min samples separated by a 2-min ISI. After a 2-min RI in a holding cage, the test phase followed, with 5 min to explore the objects specified for each procedure.

To emulate the experimental conditions in our simulations, we introduced several controlled simplifications. First, stimulus duration—that is, exposure to an object—was fixed across simulations. This departs from the experiments, where total exposure depends on the animal’s behavior and often declines with habituation. Although SOP can be extended to couple exploration time with ongoing activation, we did not implement that coupling here. Second, we assumed that the number of contexts matched the number of available objects: SOR and RR were simulated with two contexts, while OIP and RR + OIP were simulated with four. Third, within a given sample, the cumulative duration of all contexts equaled the total sample duration, and all contexts had the same length. Each object was scheduled in the second half of its context interval, so object duration was half of that context’s duration. This arrangement attempted to reflect a natural sequence in which animals first enter and explore the location and then encounter the object, yielding greater contextual exposure.

We also averaged across orders to avoid sequence effects. Arena locations were labeled as contexts C1, C2, C3, and C4. For SOR, each phase had two possible orders, C1 then C2 or C2 then C1, which produced 2 × 2 = 4 simulations in total: (C1 then C2, C1 then C2), (C1 then C2, C2 then C1), (C2 then C1, C1 then C2), and (C2 then C1, C2 then C1). By the same logic, RR used 2 × 2 × 2 = 8 simulations; OIP used 4! × 4! = 576 simulations; and RR + OIP used 4! × 4! × 4! = 13,824 simulations. Reported values are means across these runs. Finally, for the habituation stage, we simulated a single 20-min period before the first sample rather than two 10-min sessions.

With this setup in place, we present next the principal outputs of the simulations alongside reproductions of the key results from Good et al. (2007). Although both the original experiments and our simulations employed a within-subjects design, we present each task separately to improve the clarity of exposition.

**Fig. 4:**
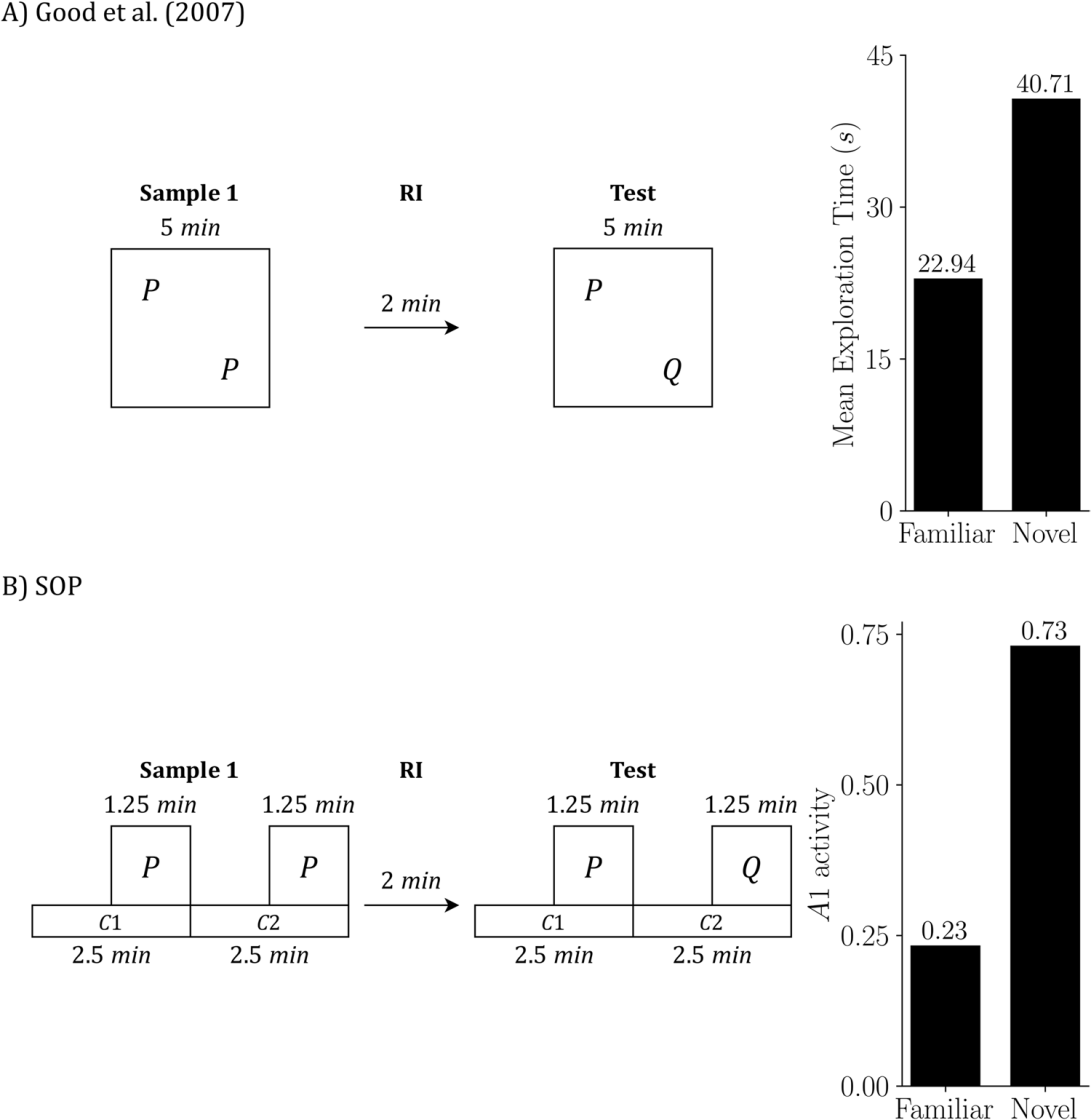
SOR: Empirical results and SOP simulation (Good et al., 2007). Empirical design and outcome from Good et al. (2007) **A)** and the matched SOP simulation **B)**. In both panels, the schedule is sample, RI = 2 min, test. In the empirical case, the sample lasts 5 min, with two identical copies of P placed in two arena locations; the animal is free to divide its exploration between these locations. In the test, the stimuli being explored are objects P and Q, each located within its respective context (physical location). In the simulation, we explicitly assign start and end times to each context and object: The sample is represented by two contexts (C1 and C2) of 2.5 min each, with P on during the final 1.25 min of each. The test is represented in the same formal way as the sample. The right bars summarize outcomes: mean exploration time (s) **A)** and predicted exploration as peak A1 **B)**, averaged across the four possible context order permutations. Empirical data adapted from Good et al. (2007)

Figure 4 focuses on SOR. Figure 4A reproduces the design and empirical outcome from Good et al. (2007): two copies of object P are sampled, a short RI follows, and the test presents familiar P against novel Q; animals explore Q more than P. Figure 4B shows the SOP simulation of the same schedule. The left side depicts the timing of contexts and object presentations used in the simulation; the right-side plots predicted exploration at test as peak A1 for P and Q, averaged across the relevant context-order permutations. Mechanistically, the novel item (Q) reaches the test with most elements inactive and can transition strongly into A1, yielding a high predicted response. The familiar item (P) arrives with elevated A2 from recent exposure (self-priming) and with additional activation from the sampled context (associatively generated priming), which leaves more elements in A2 and limits A1 at test. The simulation, therefore, reproduces the empirical novelty preference.

**Fig. 5:**
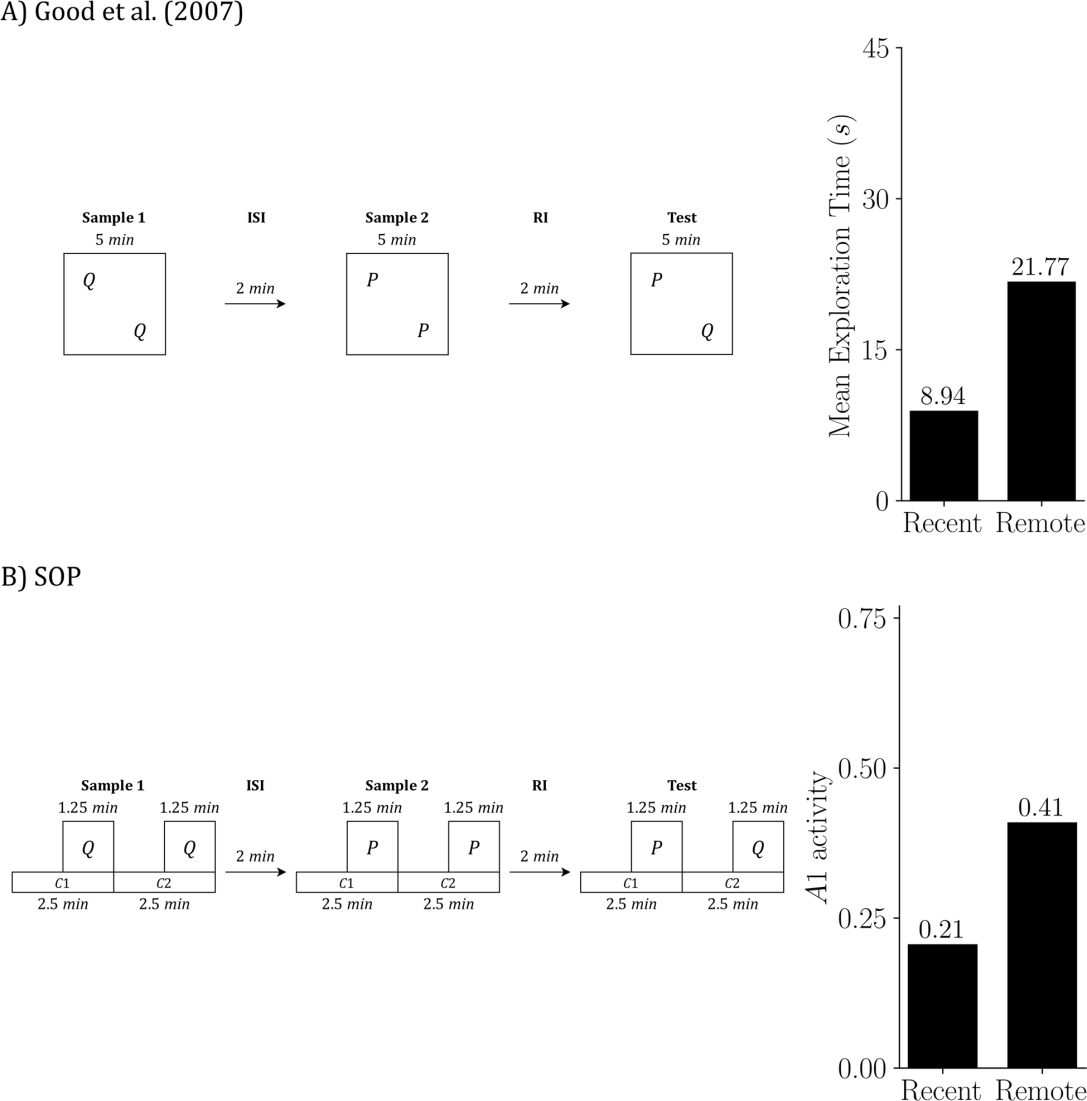
RR: Empirical results and SOP simulation (Good et al., 2007). **A)** Empirical RR design and outcome from Good et al. (2007). **B)** Matched SOP simulation. In both panels the schedule is Sample 1, ISI = 2 min, Sample 2, RI = 2 min, test. In the empirical case, Sample 1 presents two copies of Q for 5 min, Sample 2 presents two copies of P for 5 min, and the test presents P with Q for 5 min; the bars at right report mean exploration time in seconds. In the simulation, the same schedule is implemented with explicit timing: each sample comprises two contexts of 2.5 min each, with the object on during the final 1.25 min of its context; the Test is represented in the same way, with P and Q in separate contexts that begin before the objects and end with them. The key representational difference is that in **A)** contexts are locations that contain the objects, whereas in** B)** contexts are modeled stimuli that overlap with the objects and modulate their activation. Bars on the right in** B)** show predicted exploration at test as peak A1 for P and Q, averaged across the eight context-order permutations. Empirical data adapted from Good et al. (2007)

Figure 5 presents the RR task. Figure 5A shows the empirical design from Good et al. (2007): Sample 1 presents two copies of Q, then, after a short ISI, Sample 2 presents two copies of P; after a brief RI, P and Q are tested together. The bar on the right indicates that the remote item (Q) is explored more than the recent item (P). Figure 5B shows the matching SOP simulation: the left timeline mirrors the empirical schedule in simulated moments, and the right bar plot reports the predicted test outcome as peak A1. The simulation also yields remote > recent.

Why does SOP produce this pattern? As discussed earlier, both objects are sampled before the test, so both carry some A2 at the test. Because Q was sampled earlier, its self-priming has had more time to decay, leaving more elements in the inactive state and available to enter A1. In addition, during Sample 2, the contexts are active while Q is absent, which pairs context A1 with Q’s A2. This increases the inhibitory component of the context-Q link and reduces its net association, so associative priming for Q at test is weaker than for P. With both sources of priming diminished for Q, it reenters A1 more readily at test and is explored more, reproducing the empirical pattern.

**Fig. 6:**
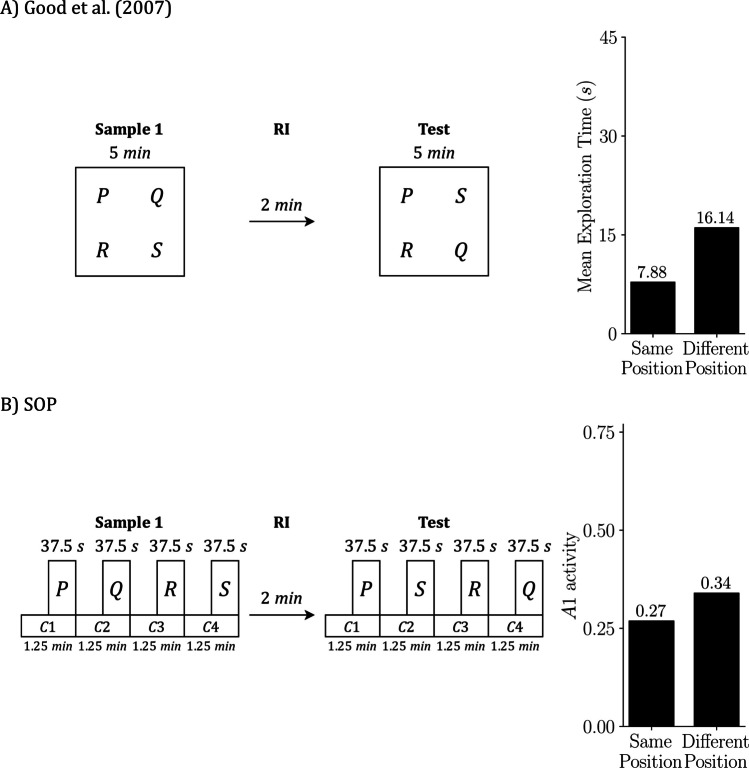
OIP: Empirical results and SOP simulation (Good et al., 2007). **A)** Empirical object in place (OIP) design and outcome from Good et al. (2007). **B)** Matched SOP simulation. In both panels the schedule is sample, RI = 2 min, test. In the empirical case, during the 5-min sample phase, four objects (P, Q, R, S) are placed in four distinct arena locations; at test (5 min), P and R remain in their original locations and Q and S are swapped; the bars at right report mean exploration time in seconds for same-location versus displaced objects. In the simulation, the same schedule is implemented with explicit timing: the sample phase is represented by four contexts labeled C1, C2, C3, C4, each lasting 1.25 min, with each object on during the final 0.625 min of its context; the test is represented in the same way, with P and R in their original contexts and Q and S exchanged. Bars on the right in **B)** show predicted exploration at test as peak A1, averaged across 576 context-order permutations (4! × 4!). Empirical data adapted from Good et al. (2007)

For OIP, Fig. 6 presents the design and outcomes for the empirical data (Fig. 6A) and the corresponding SOP simulation (Fig. 6B) from Good et al. (2007). Empirically, rats explored the displaced items (Q and S) more than the undisplaced items (P and R). In the simulation, all objects carry self-priming at test; P and R, tested in their original locations, also receive associative priming from context–object links, which keeps more elements in A2, lowers A1, and reduces exploration. Q and S, tested in new locations, lack this associative support, achieve higher peak A1, and are explored more. The model therefore reproduces both the direction and the approximate magnitude of the displacement advantage observed in the data.

**Fig. 7:**
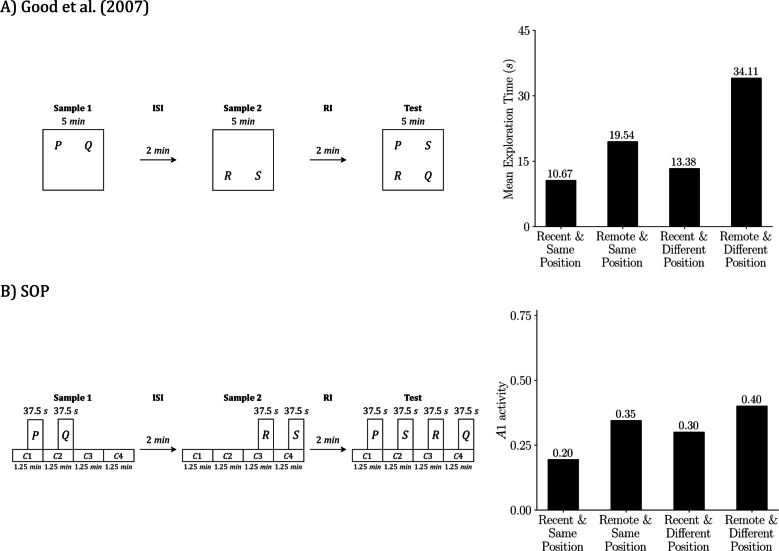
RR enhanced by OIP: Empirical results and SOP simulation (Good et al., 2007). **A)** Empirical RR enhanced by OIP (RR + OIP) design and outcome from Good et al. (2007). **B)** Matched SOP simulation. In both panels the schedule is Sample 1, ISI = 2 min, Sample 2, RI = 2 min, test. In the empirical case, Sample 1 presents P and Q for 5 min in two arena locations, Sample 2 presents R and S for 5 min in the two remaining locations, and the test presents all four objects for 5 min with P and R in their original locations and Q and S exchanged; the bars on the right report mean exploration time (s) for the four combinations of temporal status (recent vs. remote) and position (same vs. different). In the simulation, the same schedule is implemented with explicit timing: Sample 1 is represented by four contexts of 1.25 min each with P and Q on during the final 0.625 min of their contexts; Sample 2 uses the same four contexts of 1.25 min each with R and S on during the final 0.625 min of their contexts, which are different from those of P and Q; RI = 2 min; the test is represented in the same formal way, with four contexts and P and R remaining in their original contexts while Q and S are exchanged. The representational difference mirrors earlier figures: **A)** Contexts are physical locations that contain the objects, whereas in **B)**, contexts are modeled stimuli that begin before the objects, overlap with them, and end at the same time. Bars on the right in **B)** show predicted exploration at test as peak A1, averaged across the 13,824 context-order permutations (4! orders in Sample 1 × 4! in Sample 2 × 4! at test). Empirical data adapted from Good et al. (2007)

Figure 7 summarizes the RR + OIP procedure. Empirically (Fig. 7A), exploration depends on both temporal status and position: the remote-and-displaced item (Q) is explored the most, the recent-and-non-displaced item (R) the least, and the other two conditions (remote–same position and recent–different position) fall in between, with no reliable difference reported. The SOP simulation (Fig. 7B) reproduces this ordering. Q was sampled earlier, so its self-priming has more time to fade, leaving more elements available to enter A1 at test. During Sample 2 the contexts are active while Q is absent, which weakens the net context–Q association and reduces associative priming for Q at test. Q is also displaced, so its current context provides no associative support. Together, these factors yield the highest peak A1 for Q. In contrast, R is recent and nondisplaced, so both self-generated and associative priming are strong, suppressing A1 and resulting in the lowest exploration. The remaining conditions land between these extremes, matching the qualitative pattern in the data.

Across Good et al.’s (2007) four procedures, the SOP simulations captured the canonical patterns with a fixed parameter set: in SOR, novel > familiar; in RR, remote > recent; in OIP, displaced > same-location; and in RR + OIP, remote–displaced highest and recent–same lowest, with the other two conditions intermediate. Predicted outcomes, expressed as peak A1 (our exploration proxy), closely matched the qualitative ordering and relative magnitudes reported empirically in Figs. 4, 5, 6, and 7.

## Simulating delay effects (Tam et al., 2014)

Our second target study, Tam et al. (2014), examined how delays shape performance in SOR, RR, and OIP for sham-operated and lesioned rats. Here, “delay” means the sample-to-test interval for SOR and OIP (RI) and the interval between the two samples for RR (ISI). We simulate the sham group to assess SOP’s baseline predictions at two delays: 5 min and 120 min.

Procedurally, each rat completed two 10-min habituation sessions, followed by two blocks of recognition trials in fixed order. Block 1 presented SOR, then RR (temporal order), then OIP. Block 2 presented OIP, then RR, then SOR. Delay assignments were counterbalanced within subjects: half of the animals experienced 5 min in Block 1 and 120 min in Block 2, and the remainder the reverse. Each SOR and OIP trial used one 5-min sample; RR used two 5-min samples. All tests lasted 3 min. Our simulations follow these timings and task structures, using the same modeling simplifications as in the Good et al. (2007) section.

As before, we present each task separately for clarity, even though both the experiments and our simulations used a within-subjects design.

**Fig. 8:**
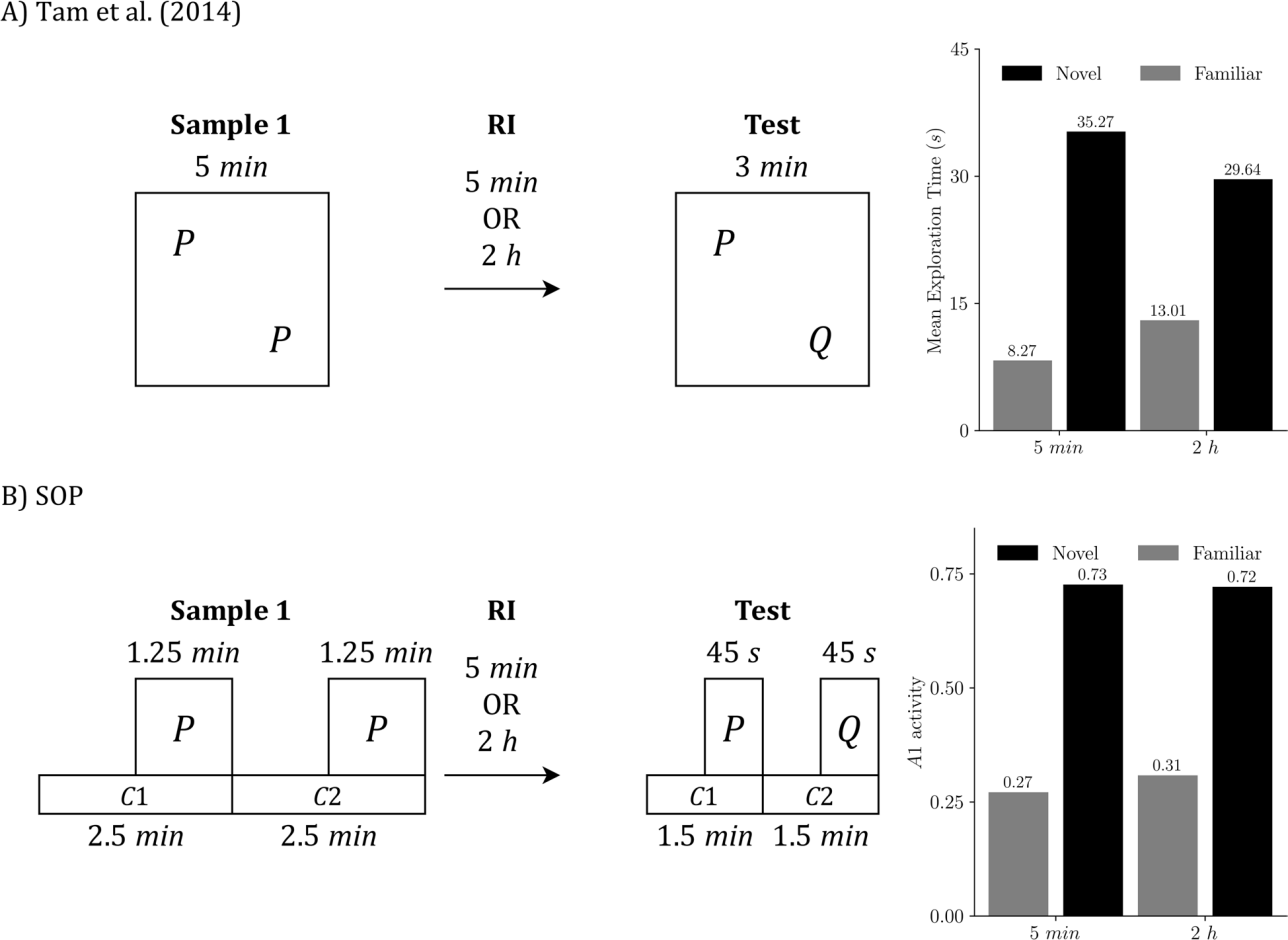
SOR under two RIs: Empirical results and SOP simulation (Tam et al., 2014). **A)** Empirical SOR design and outcome from Tam et al. (2014). **B)** Matched SOP simulation. Both panels use the schedule sample, RI, test with RI = 5 min or 120 min, and all times are in minutes. In the empirical case, the sample presents two copies of P for 5 min, the test presents familiar P with novel Q for 3 min, and the bars on the right report mean exploration time in seconds by delay. In the simulation, the same schedule is implemented with explicit timing: the sample phase is represented by two contexts of 2.5 min each with P on during the final 1.25 min of each; the test is represented in the same way with two contexts of 1.5 min each and P and Q on during the final 0.75 min. The only representational difference is how contexts are instantiated:** A)** they are locations that contain the objects, whereas in **B)** they are modeled stimuli that begin before the objects, overlap with them, and end at the same time. Bars on the right in **B)** show predicted exploration at test as peak A1 for P and Q, averaged across the four possible context-order permutations. Empirical data adapted from Tam et al. (2014)

Figure 8 illustrates the SOR procedure from Tam et al. (2014). Figure 8A presents the empirical design and outcome,^3^ while Fig. 8B displays the matching SOP simulation. In both, the familiar object is explored more after a longer RI, which reduces the novelty preference. In SOP terms, at 5 min, the familiar item is strongly primed by both self-generated and associative mechanisms, keeping many elements in A2 and yielding a low A1 at test. At 2 hours, the self-generated component has largely decayed, so only associative priming remains; more elements are available to enter A1, and exploration rises. The novel item, whose elements start in I, reaches the same peak A1 regardless of the delay in the simulation. Empirically, novelty is still preferred at both delays, though the advantage is smaller at 2 hours.

**Fig. 9:**
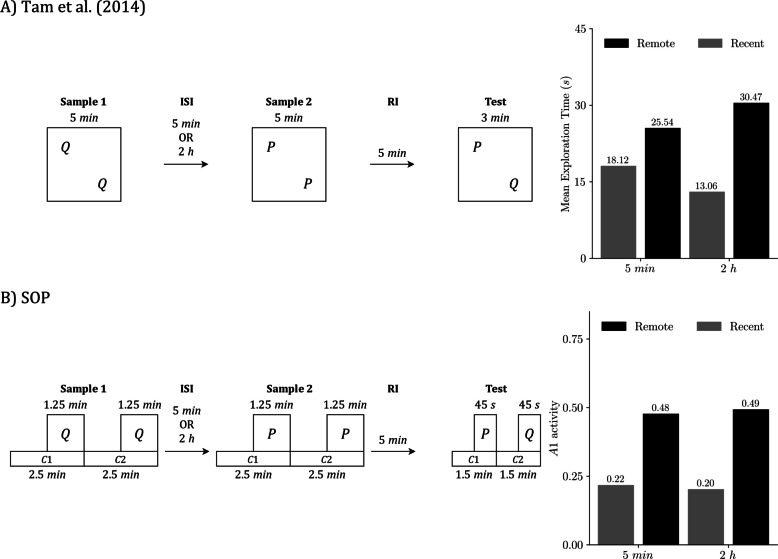
RR under two ISIs: Empirical results and SOP simulation (Tam et al., 2014). **A)** Empirical RR design and outcome from Tam et al. (2014). **B)** Matched SOP simulation. Both panels use the schedule: Sample 1, ISI = 5 min or 120 min; Sample 2, RI = 2 min; test. In the empirical case, Sample 1 presents two copies of Q for 5 min, Sample 2 presents two copies of P for 5 min, and the test presents P with Q for 3 min. The bars at the right report the mean exploration time in seconds at each ISI. In the simulation, the same schedule is implemented with explicit timing: each sample comprises two contexts of 2.5 min each, with the object on during the final 1.25 min of its context; the test is represented in the same way with two contexts of 1.5 min each of P and Q on during the final 0.75 min. The representational difference lies in that in** A)**, contexts are represented as arena locations that contain the objects, whereas in **B)**, contexts are modeled as stimuli that precede the objects, overlap with them, and conclude simultaneously. Bars on the right in** B)** show predicted exploration at test as peak A1 for P and Q, averaged across the eight possible context order permutations. Empirical data adapted from Tam et al. (2014)

Figure 9 summarizes the RR task under two ISIs. Empirically (Fig. 9A), the remote item was explored more than the recent item at both intervals, and the difference was larger at the 2-hour ISI. In our SOP simulations (Fig. 9B), the remote item’s predicted exploration was essentially unchanged across ISIs, while the recent item showed lower exploration at the longer ISI; the remote > recent contrast therefore also increased at 2 hours. The divergence in how the remote bar changes may arise from measurement: the experiment uses a mutually exclusive choice test, so time spent on one object reduces time available for exploring the other, whereas our simulations use fixed presentation durations and treat peak A1 during each object’s presentation as proportional to exploration. Despite this difference, both sources indicate a greater RR effect when the samples are separated by 2 hours.

**Fig. 10:**
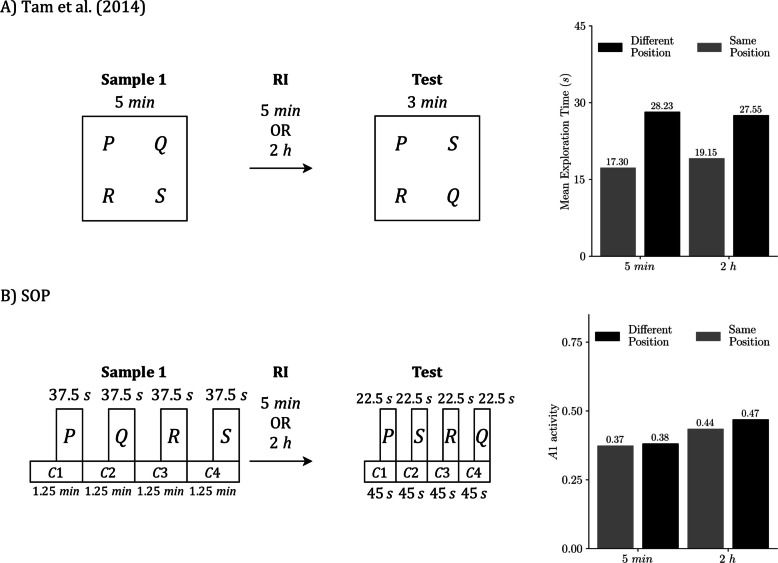
OIP under two RIs: Empirical results and SOP simulation (Tam et al., 2014). **A)** Empirical object in place (OIP) design and outcome from Tam et al. (2014). **B)** Matched SOP simulation. Both panels use the schedule sample, RI = 5 min or 120 min, test. In the empirical case, the sample presents four objects (P, Q, R, S) in four distinct arena locations for 5 min; the test lasts 3 min, with P and R in their original locations and Q and S swapped. Bars on the right report the mean exploration time in seconds for displaced versus same-location objects at each delay. In the simulation, the same schedule is implemented with explicit timing: the sample is represented by four contexts (C1–C4) of 1.25 min each, with each object on during the final 0.625 min of its context. The test is represented in the same way, divided equally across four contexts of 0.75 min each, with the object on during the final 0.375 min. The representational difference is how contexts are instantiated: in **A)** they are physical locations that contain the objects, whereas in **B)** they are modeled stimuli that begin before the objects, overlap with them, and end at the same time. Bars on the right in **B)** show predicted exploration at test as peak A1, averaged across the 576 context-order permutations (4! in sample × 4! in test). Empirical data adapted from Tam et al. (2014)

Figure 10 presents the OIP procedure. Empirically (Fig. 10A), rats explored displaced items more than undisplaced items, and the magnitude of that displacement advantage did not differ reliably between the 5-min and 2-h RIs. The SOP simulation (Fig. 10B) reproduces this ordering, although the displacement effect is more modest under both delays with the current parameterization. This is because associative priming from the test context is weak, so the difference between same location and displaced items remains small. More importantly, because the RI occurs outside the experimental arena, there is no extinction of the context-to-object associations formed in the sample phase. The only systematic change across delays is the decay of self-priming; after 2 h, more elements have left A2, allowing larger A1 at test for all items and producing a uniform elevation of predicted exploration while preserving the displaced > same location pattern.

Overall, SOP fits the Tam et al. data well across delays. In SOR (Fig. 8), the model reproduces the reduced novelty preference at the 2-h RI; in RR (Fig. 9), it yields a stronger remote > recent contrast at the longer ISI; and in OIP (Fig. 10), it preserves the displaced > same-location advantage at both delays while predicting a modest elevation in overall exploration at 2 h. Aside from minor discrepancies traceable to measurement assumptions (choice time vs. fixed presentation and peak A1), the simulations closely match the empirical ordering and relative magnitudes.

## Simulating effects of the interval between samples: Whitt and Robinson (2013)

**Fig. 11:**
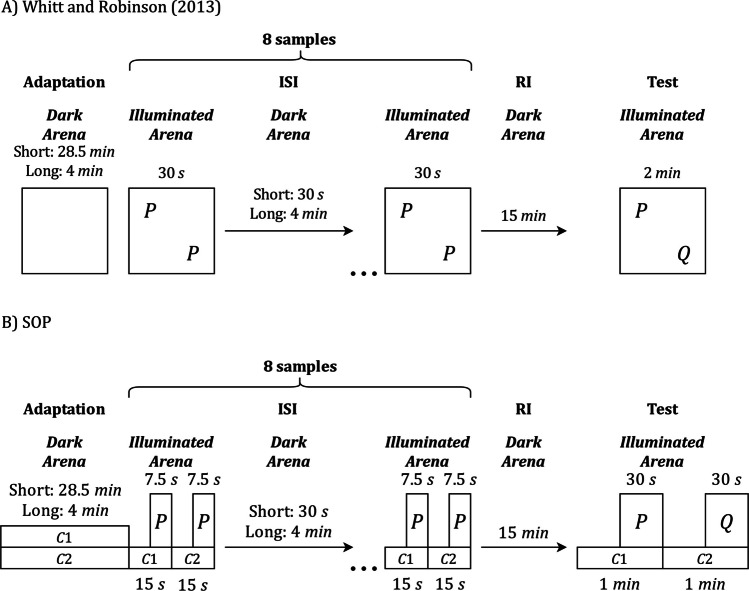
ISI-Effects with multiple samples in SOR: Experimental and SOP simulation designs (Whitt & Robinson, 2013). **A)** Empirical SOR procedure reported by Whitt and Robinson (2013). **B)** Matching SOP simulation of that procedure. In both panels, the overall schedule is the same: eight 30-s samples of familiar P separated by either a short ISI of 30 s or a long ISI of 4 min, followed by a 15-min RI and a test with familiar P and novel Q. The main difference lies in how sampling and context are represented. In the empirical panel, each 30-s sample provides two identical copies of P in two arena locations, and the animal is free to divide its exploration across those locations. For present purposes, these locations are treated as the relevant contexts. Because the rats could use those positional cues only when the arena was illuminated, context in this figure refers specifically to illuminated spatial locations. The general dark state of the arena was common to all conditions, objects, and intervals and was therefore omitted, both to simplify the representation and to maintain consistency with the description of the other experiments. In the simulation panel, the same 30-s illuminated sample is rendered as two 15-s context periods, one for each location, with P active during the final 7.5 s of each period and the preceding 7.5 s representing the context alone. Thus, each sample contains exposure to P in both contexts while preserving a simple context structure for the model. The test is represented in the same way, with one context period for P and one for Q. Simulated summaries are averaged across the 2^9^ = 512 possible context-order permutations

Our following target study is Whitt and Robinson (2013), who investigated the effect of the interval between samples, or the inter-sample interval ISI effect in SOR using a within-subject design. The animals were tested in arenas in which the stimuli were placed inside glass cylinders. During stimulus presentations, the cylinders were illuminated, allowing visual access to the objects, whereas between presentations the room remained dark, providing a more general contextual background. Before the recognition phase, all rats received a pretraining phase in which they were exposed to the experimental context for durations matched to the later massed and spaced procedures. This phase served to familiarize the animals with the arena and the lighting regime. After pretraining, all rats received both a massed condition and a spaced condition, presented in counterbalanced order and separated by 3 days. In one condition, the familiar object, which we denote here as P, was presented on both sides of the arena for eight 30-s samples separated by 30 s. In the other, the same sampling sequence was used, but the interval between samples was 4 min. After each sampling session, the animals spent 15 min outside the experimental context and then received a test in which familiar P was presented on one side of the arena and a novel object, Q, on the other. Exploration was assessed from the animal’s approach to the two objects. Figure 11A provides a simplified schematic illustration of this empirical design, retaining the core temporal structure needed for the matching SOP simulation.

Figure 11B shows the corresponding SOP implementation of this simplified design. The model preserves the same overall temporal structure, including the initial period before the first sample, the eight sample presentations separated by either a short ISI of 30 s or a long ISI of 4 min, the 15 min RI, and the final choice test. Arena locations are represented as contexts C1 and C2. Within each sample, the context window lasts 1 min, and object P is active during the final 30 s, reflecting that, in the empirical procedure, visual access to the object was restricted to the illuminated portion of each sample. At test, the model includes one 1-min context period with familiar P and one 1-min context period with novel Q, for a total of 2 min. Predicted outcomes are expressed as peak A1 activation for familiar and novel objects, averaged across all permutations of context order within the sample sequence and at test.

**Fig. 12:**
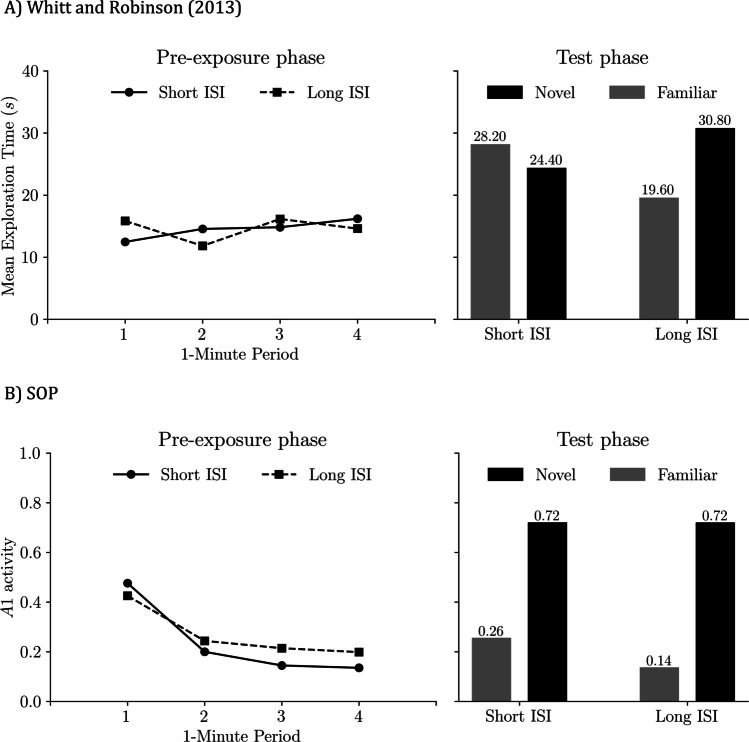
Exploration under short versus long ISIs in multi-sample SOR: Empirical results and SOP simulation (Whitt & Robinson, 2013). **A)** (Whitt & Robinson, 2013) shows, on the left, mean exploration of the to-be-familiar object across successive 1-min bins during preexposure for the short ISI (30 s) and long ISI (4 min) schedules; the bars on the right show test exploration for novel and familiar objects. **B)** Matched SOP simulation: the left plot gives predicted A1 activity during preexposure for the two ISIs, and the right bars give predicted test outcomes as peak A1 (our exploration proxy) for novel and familiar objects. Empirical data adapted from Whitt and Robinson (2013)

Figure 12 summarizes the results reported by Whitt and Robinson (2013) alongside the SOP simulation. During the preexposure phase (left plots), the empirical traces for short and long ISIs show no sustained separation: they cross and settle to similar exploration levels. By contrast, the SOP simulation predicts a clearer divergence. With a short ISI, closely spaced samples create stronger self-priming, keeping more elements in A2 and producing a steeper decline in responding. The long ISI allows for more recovery time between samples and sustains higher responses.

In the test phase (right plots), both sources agree that the long ISI schedule yields a larger novelty preference. In SOP, the critical difference between ISIs is associative rather than self-generated, given the long RI: with a long ISI, the familiar item’s A1 during sampling is less suppressed, so there are more A1_context_–A1_object_ co-occurrences and stronger excitatory context-object links, which later reduce re-entry to A1 at test. With a short ISI, stronger SGP during sampling dampens the object’s A1, yielding fewer A1–A1 overlaps and weaker associative support at test. A slight discrepancy remains in the short-ISI condition, where the empirical bars show a nonsignificant trend of familiar > novel, whereas SOP predicts novel > familiar under our assumptions. This likely reflects factors not modeled here, such as using an even shorter ISI that prevents effective context–object associations from developing.

## Simulating ISI × RI manipulations in Y-maze SOR: Sanderson and Bannerman (2011)

Our final simulation targets a study by Sanderson and Bannerman (2011, Experiment 1), who manipulated both the ISI during sampling and the RI before test in a Y-maze version of SOR without physical objects in mice. During sampling, each mouse completed 10 visits: on each visit, it started in the START stem and could enter one open goal arm while the other arm was blocked. Visits were separated by either a short ISI (1 min) or a long ISI (24 h). At test, after either a short RI (1 min) or a long RI (24 h), both arms were open for 2 min to assess exploration of the familiar versus the novel arm.

**Fig. 13:**
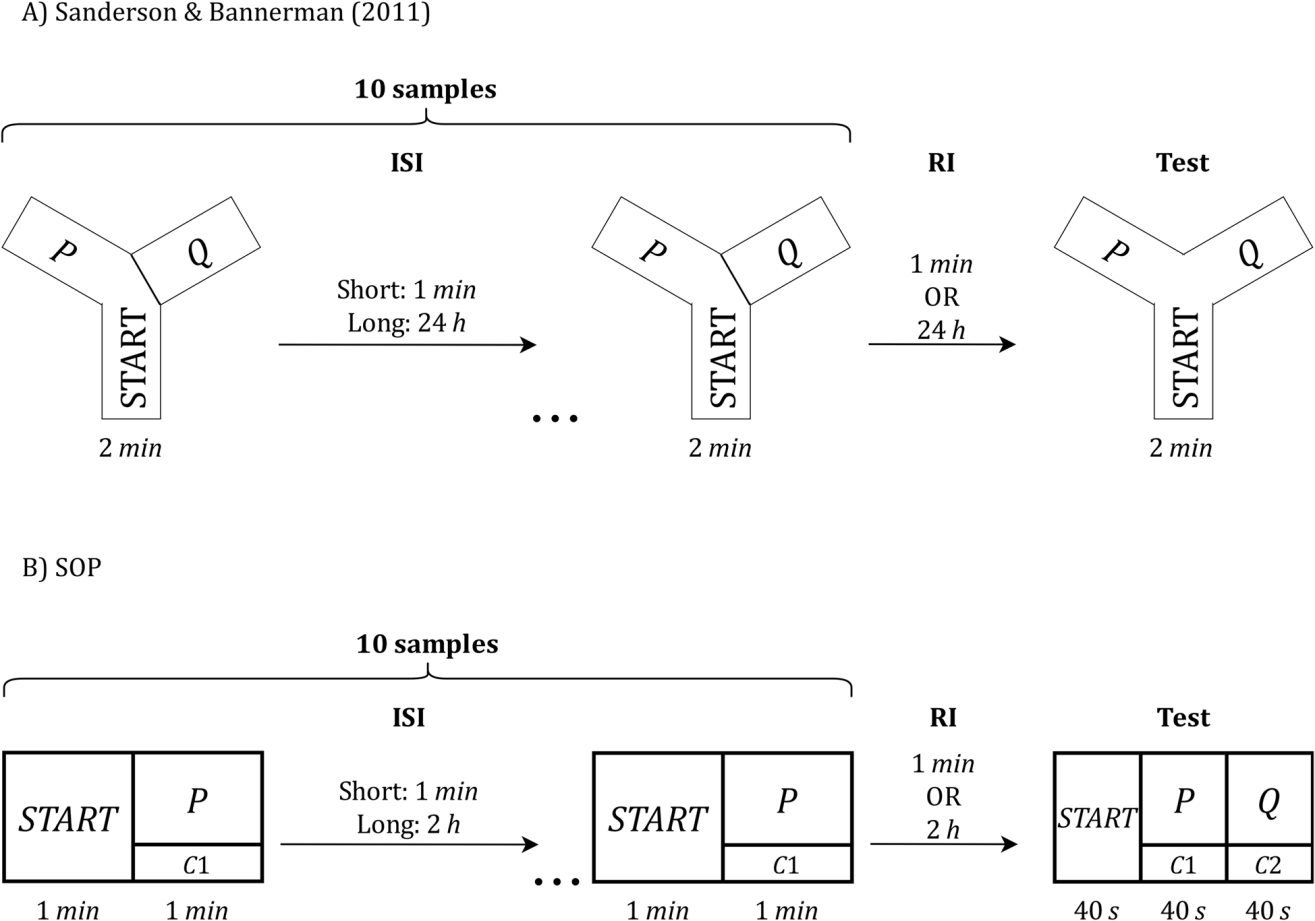
Design of empirical and simulated Y-maze task manipulating ISI and RI (Sanderson & Bannerman, 2011). **A)** Empirical Y-maze SOR design from Sanderson and Bannerman (2011, Experiment 1). **B)** SOP simulation of that design. In both, sampling consists of 10 visits from the START stem, with access to one open goal arm per visit while the other arm is blocked; visits are separated by either a short ISI = 1 min or a long ISI (empirical 24 h; simulated 2 h for efficiency, which is sufficient for complete decay of self-priming). At test, after an RI that is short (1 min) or long (empirical 24 h; simulated 2 h), both arms are open for 2 min to compare exploration of the familiar versus the novel arm. Because the study used locations rather than discrete objects, the simulation treats the unconditioned event and its context as coextensive in time. The START context is always presented first on each sample and at test; counterbalancing occurs only at test by permuting which arm is familiar versus novel

Figure 13 shows the empirical design and our simulation design approach. Because this experiment used locations rather than discrete objects, we adopted several adaptations. First, since animals explored an area, the unconditioned event and its context were treated as coextensive in time. Second, the START stem was always presented first on each sample and during the test, so counterbalancing occurred only at the test by permuting which arm was familiar versus novel. Third, because the study quantified sampling by entry frequency into the familiar arm, we assumed peak A1 scales with both exploration time and entry count; for simplicity, we modeled one entry per sample. Fourth, for computational efficiency, we set the long ISI and long RI to 2 h rather than 24 h. Self-priming in the model dissipates totally within 2 h, so this shorter delay yields equivalent qualitative outcomes.

**Fig. 14:**
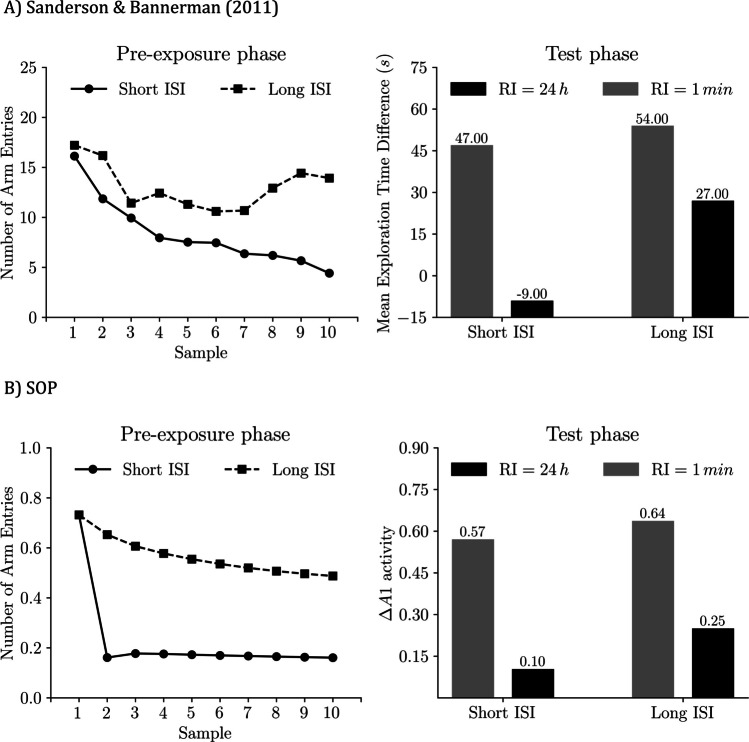
Y-maze SOR: Effects of ISI and RI: Empirical results and SOP simulations (Sanderson & Bannerman, 2011). The left-hand side of **A)** presented the approximate values of the mean exploration time of the familiar arm in both massed and distributed conditions. The left-hand side of **B)** displays the maximal predicted exploration according to simulations of the SOP model in both massed and distributed conditions. The right-hand side of each plot displays bars representing the difference in exploration between novel and familiar arms in the massed and distributed conditions with two different RIs, in empirical and simulated data. To match the study’s metric, the simulation assumes one entry per visit and interprets peak A1 as scaling with both exploration time and entry count. Empirical data adapted from “Competitive Short-Term and Long-Term Memory Processes in Spatial Habituation,” by D. Sanderson and D. Bannerman, 2011, *Journal of Experimental Psychology: Animal Behavior Processes, 37*(2), pp. 192–193 (10.1037/a0021461). Copyright 2011 by the American Psychological Association

Figure 14 shows empirical and simulated results from Sanderson and Bannerman (2011, Experiment 1). On the left of each panel are sampling-phase data: mean entries into the to-be-familiar arm across the 10 samples, averaged over RI-groups. In both the data and the model, the first sample looks similar for the short ISI and long ISI conditions. Thereafter, entries decline faster with the short ISI than with the long ISI, consistent with stronger self-priming when visits are closely spaced.

On the right of each panel are test-phase outcomes: novelty preference expressed as novel-familiar (empirical: exploration time; simulation: peak A1). With a 1-min RI, novelty preference is larger in both ISI conditions because the familiar arm is still affected by both self-generated and associatively generated priming, which suppresses its A1 and hence exploration. With a long RI (24 h in the data; 2 h in the simulations), self-priming has dissipated, leaving only associative influences, resulting in a smaller novelty preference.

Furthermore, the model correctly predicted that animals trained in the long ISI condition showed greater preference for the novel arm compared with those trained with a 1-min ISI. Similar to Tam et al. (2014), in the short ISI condition, strong SGP during the preexposure phase resulted in temporal concurrence between the A2 state of the familiar arm and the A1 activity of the contexts. Consequently, the association between the context and the arm (V_C1-P_) was predominantly influenced by an inhibitory learning process, thereby attenuating the effectiveness of AGP in the test phase. Conversely, in the long ISI condition, the A1 states of the context and the familiar arm co-occurred, fostering excitatory associative learning and therefore, enhancing AGP efficacy during the test.

## Quantitative assessment of model predictions

To complement our previous results, we quantified how closely SOP reproduces effect magnitudes using a single, task-general mapping from the model’s latent output to exploration time. Because we do not optimize model parameters, the resulting error metrics directly reflect the extent to which observed magnitudes emerge from the model’s general mechanics under canonical parameterization, rather than from post hoc tuning to individual datasets.

The central problem in this quantitative comparison is one of measurement scale. SOP’s simulations yield an object-level test output in arbitrary units of A1 activity, whereas the empirical studies report object exploration in seconds. We therefore specify an explicit observation model that links the model’s latent response to the measured behavioral unit. Following our assumption that the peak A1 of object *i* is proportional to the total time spent exploring it, we implemented a simple linear scaling:

$${\widehat{y}}_{i }\mathrm{=} k{\widehat{A1}}_{i},$$

where ${\widehat{y}}_{i}$ is the predicted exploration time in seconds for *i*, ${\widehat{A1}}_{i}$ is the model output for that object in the test, and *k* is the global scaling constant that converts A1 activation units into seconds. We estimate *k* using weighted least squares (WLS) on all individual object predictions. First, each object was weighted by the inverse variance of its estimate to account for differences in measurement precision.^4^ Second, these weights were normalized to avoid bias toward studies that report more object exploration level means and toward tasks that contribute more objects. The scaling constant *k* was then estimated as:

$$k \mathrm{=}\frac{{\sum}_{i}{w}_{i}{\widehat{A1}}_{i}{y}_{i}}{{\sum}_{i}{w}_{i}{\widehat{A1}}_{i}^{2}},$$

where *y*_*i*_ is the observed exploration time for object *i* and *w*_*i*_ is the final normalized weight.

Using this single global mapping, we obtained exploration level predictions for object in seconds (${\widehat{y}}_{i}$) and residuals (${e}_{i} \mathrm{=} {\widehat{y}}_{i}-{y}_{i}$). The estimated scaling constant was k = 55.82, with a 95% exact study-level bootstrap confidence interval of [45.58, 65.07] obtained by enumerating all 256 resamples. A leave-one-study-out (LOSO) analysis revealed moderate sensitivity to study composition: removing Sanderson and Bannerman (2011) from dataset reduced k by approximately 13%, while removing Whitt and Robinson (2013) increased *k* by approximately 8%. Omitting Good et al. (2007) or Tam et al. (2014) produced minimal changes (<3%).

As shown in Table 1, under the linear scaling, object-level predictions varied across studies. Good et al. (2007) and Tam et al. (2014) showed the closest correspondence in magnitude, with relatively small absolute errors and slightly positive bias. In contrast, Sanderson and Bannerman (2011) exhibited the largest discrepancies, characterized by systematic underprediction, whereas Whitt and Robinson (2013) showed large absolute deviations despite a comparatively small net bias. When pooled across all objects, the model displayed a slight overall tendency to underpredict exploration times, with moderate absolute error and almost 40% of the variance captured. These patterns suggest that the global scaling *k* provides a reasonable common mapping to seconds, while highlighting residual between-study heterogeneity.

**Table 1:** Object-level prediction error in exploration time (seconds) by study

Study	N° of objects		wBias	wMAE	wRMSE	wR^2^
Good et al. (2007)	10	0.54		4.36	5.61	
Tam et al. (2014)	12	1.60		4.62	5.29	
Whitt & Robinson (2013)	4	−1.78		13.14	13.31	
Sanderson & Bannerman (2011)	8	−7.90		12.34	15.27	
Overall	34	−1.89		8.62	10.84	0.388

Metrics summarize object-level prediction error for mean exploration times after mapping SOP output to seconds under a global scaling k = 55.82. All metrics are in seconds and were weighted to give equal influence to each study and task. Study-level wR2 is not reported due to instability with small numbers of objects. N° of objects = number of individual objects means; wBias = weighted mean bias; wMAE = weighted mean absolute error; wRMSE = weighted root mean squared error; wR^2^ = weighted coefficient of determination.

We next evaluated model performance at the level of empirically reported contrast, which provides a more direct test of effect magnitude. For each experimental condition, we computed the empirical contrast Δy in seconds as the difference between object Q and object P exploration means in test, with Q being the novel/remote/undisplaced object and P being the familiar/recent/in-place object. Correspondingly, we also calculated the SOP contrast $\Delta \widehat{A1} \mathrm{=} {\widehat{A1}}_{Q}-{\widehat{A1}}_{P}$ in model activity units from the simulated object outputs at test. Predicted contrasts were then obtained in seconds as $\Delta \widehat{y} \mathrm{=} k\Delta \widehat{A1}$, and contrast residuals were defined as ${e}_{\Delta } \mathrm{=} \Delta \widehat{y}-\Delta y$. Contrast-level accuracy was summarized using the same weighted bias, MAE, and RMSE metrics as in the object-level analysis, reported both overall and by study in Table 2.^5^

**Table 2:** Contrast-level prediction error in exploration time (seconds) by study

Study	N° of contrasts	wBias	wMAE	wRMSE	Sign acc.
Good et al. (2007)	6*	0.29	5.25	6.55	100%
Tam et al. (2014)	6	−1.04	5.55	6.43	100%
Whitt & Robinson (2013)	2	25.56	25.56	25.90	50%
Sanderson & Bannerman (2011)	4	−7.96	15.36	15.48	75%
Overall	18*	4.21	12.93	15.77	81.2%

Metrics summarize contrast-level prediction error after mapping SOP output to seconds under a global scaling k = 55.82. All metrics are in seconds and were weighted to give equal influence to each study and task. The asterisk (*) indicates that the contrast count includes three component contrasts from a single task (RR + OIP in Good et al., 2007), with the total weight of that procedure split equally across the three contrasts. N° of contrasts = number of contrasts for study; wBias = weighted mean bias; wMAE = weighted mean absolute error; wRMSE = weighted root mean squared error; Sign Acc. = percentage of correctly predicted signs.

As shown in Table 2, contrast-level performance was heterogeneous across studies. Good et al. (2007) and Tam et al. studies showed once again the strongest agreement, with consistently correct directional predictions and comparatively small magnitude errors. In contrast, Sanderson and Bannerman and Whitt and Robinson experiments produced the largest discrepancies. In these studies, the short-ISI conditions (and long-RI condition in Sanderson and Bannerman) exhibited greater empirical exploration for familiar than for novel objects. Under the canonical SOP parameterization and our observation assumption that exploration is proportional to peak A1 activation, such reversals are not reproduced, since priming mechanisms predict reduced novelty-driven exploration for previously sampled objects. These mismatches therefore likely reflect study-specific factors outside the scope of the present simplified simulations and observation model, rather than limitations that could be resolved by perturbations of the parameter set.

In general, the model correctly predicted the direction of most empirical contrasts, although magnitude errors were larger at the contrast level than at the object level. The positive overall bias indicates a net tendency to overestimate effect magnitudes, driven primarily by the large discrepancies observed in Whitt and Robinson (2013). We did not report wR^2^ in this case due to the small number of contrasts, which renders this metric unstable and uninformative.

Taken together, the quantitative assessment indicates that, under a single global observation mapping from SOP output to raw exploration seconds and without any parameter optimization, our implementation captures the direction of most recognition effects and yields a reasonable baseline account of effect magnitudes. However, this analysis also revealed systematic discrepancies that delimit the scope of the present approach. A key limitation is that a single linear scaling constant cannot absorb between-study and between-task differences that alter baseline exploration levels and measurement practices, including test duration, apparatus constraints, and scoring practices, among others. For example, in Sanderson and Bannerman’s (2011) Y-maze procedure, exploration is operationalized as arm preference rather than interaction with test objects, and the mapping from A1 peak to seconds is therefore only an approximation to a different behavioral readout. Despite these limitations, the present quantitative benchmark is informative because it identifies which effect magnitudes emerge from SOP’s core priming dynamics and delineates where mismatches likely reflect procedural factors outside the scope of the present simulations, all under a task-general observation model with fixed parameters and no dataset-specific optimization.

Robustness to parameter variation and the theoretical sources of variability in predicted outcomes are evaluated in the subsequent section on global and local sensitivity analyses.

## Global and local sensitivity analyses of SOP theoretical processes

To assess whether our predictions arise from the core dynamics of SOP rather than from arbitrary parameterization, we conducted sensitivity analyses at both global and local scales, focusing on the model’s theoretical processes. The global component combines a qualitative mapping of outcome patterns with a quantitative decomposition of predictive variability, while the local component provides an interpretable, directional assessment of how individual parameters modulate the predicted contrasts. Across all analyses we focused on the same four parameters most relevant to self-generated and associatively generated priming: pd1, pd2, L+, and L− sampled log-uniformly over [0.001, 1].

As a first global, qualitative analysis, we applied parameter space partitioning (PSP; Pitt et al., 2006) to identify distinct outcome signatures across tasks and to estimate the relative volume of parameter regions supporting each pattern under increasingly stringent contrast magnitude thresholds (ε). For each sampled parameter set, task-level outcomes were classified as robust, weak, or fail based on whether the predicted contrast exceeded a proportional criterion. As in the quantitative assessment section, let ${\widehat{A1}}_{Q}$ and ${\widehat{A1}}_{P}$ denote the peak A1 activity during the test for the two objects being contrasted, with Q being the novel/remote/displaced object and P being the familiar/recent/in-place object. An effect was labeled robust when ${\widehat{A1}}_{Q }- \left(1 \mathrm{+} \varepsilon \right){\widehat{A1}}_{Q }\mathrm{>} 0$, evaluated at $\varepsilon \in \{0.05, 0.10, \, 0.15\}$; weak when it preserved only the ordinal relation ${\widehat{A1}}_{Q}- {\widehat{A1}}_{P} \mathrm{>} 0$; and fail when the ordinal relation was violated or when both responses show nonsignificant activity, operationalized as $max({\widehat{A1}}_{Q}, \, {\widehat{A1}}_{P})\le 0.005$. Table 3 reports the resulting corrected volume proportions for each outcome level. This exploration spans the full mathematically admissible parameter space, including regimes that may be psychologically implausible or pathological (e.g., pd1 < pd2 or L+ < L−). These results should therefore be interpreted as a comprehensive robustness assessment across all feasible configurations that were sampled.

**Table 3:** Corrected volume proportions of qualitative outcomes of PSP across tasks and robustness thresholds

Outcome	Task	Robustness criterion		
		ε = 0.05	ε = 0.10	ε = 0.15
Robust	SOR	72.5% (2.6)	62.9% (1.9)	57.9% (2.2)
	RR	37.2% (1.2)	34.7% (1.3)	32.3% (1.1)
	OIP	12.7% (2.0)	8.6% (1.3)	6.6% (2.1)
	All	6.7% (0.5)	4.5% (0.6)	3.3% (0.3)
Weak	SOR	26.3% (1.4)	35.8% (1.5)	41.5% (2.8)
	RR	21.7% (1.6)	25.4% (3.6)	28.4% (2.7)
	OIP	16.9% (0.8)	21.5% (2.0)	23.9% (1.4)
Fail	SOR	1.3% (1.7)	1.3% (1.8)	0.6% (1.4)
	RR	41.0% (1.8)	39.9% (3.1)	39.3% (3.2)
	OIP	70.3% (1.8)	69.9% (2.7)	69.5% (1.5)

Values are mean-corrected volume percentages across five PSP runs. Parentheses report standard deviation across runs. “Robust” denotes satisfaction of the proportional criterion with threshold ε $\in \{0.05, 0.10, \, 0.15\}$; “Weak” denotes preservation of the ordinal relation; “Fail” denotes violation of the ordinal relation or negligible responding 0.005 in any of both objects. SOR = spontaneous object recognition; RR = relative recency; OIP = object-in-place.

PSP revealed that the model’s qualitative predictions vary markedly in their robustness across tasks. For SOR, the predicted pattern emerges as a broad consequence of SOP’s dynamics: the vast majority of parameter configurations within the corrected volume produce greater exploration of the novel object than the familiar object, with robust effects dominating across all threshold levels and very few failures. As stringency increases, of those parameter set that transition, most go from robust to weak classification rather than failing outright. This stability indicates that the novelty preference emerges naturally from SOP mathematical architecture instead of depending on parameter alignment.

For RR, qualitative support is less pervasive but still substantial. A meaningful proportion of the corrected volume yields the expected ordering remote > recent, and the robust fraction remains relatively stable across threshold levels, with a corresponding shift toward weak classification as the criterion increases. Failures are more common than for SOR, indicating that RR depends more strongly on the balance between parameters, but the effect is nonetheless supported across a broad region of admissible settings.

In contrast, OIP constitutes a more demanding prediction, exhibiting greater sensitivity to parameter configuration. Robust effects arise in a smaller, more constrained region of the corrected volume, and failures dominate across thresholds. This means that displaced > in-place arises in a more specific alignment of decay dynamics and associative influence. Achieving this differential effect demands that associative learning be strong enough to form meaningful context-object bindings during preexposure sufficient to decrease exploration for the in-place object, while decay parameters must allow the displaced object to recover its exploratory response at test in the absence of context-object association.

Requiring all three tasks to simultaneously exhibit robust effects identifies a nontrivial yet bounded region of parameter space. Although this subset is small, its persistence across all threshold levels confirms that coherent multi-task performance is possible within SOP when parameters are appropriately coordinated to balance the computational demands of each paradigm.

As summarized in Table 4, the centroid and dispersion (geometric mean and geometric standard deviation, respectively) of parameters supporting simultaneous robust outcomes reveal a consistent structure across threshold levels. Notably, the parameter values adopted throughout this manuscript (pd1 = 0.1, pd2 = 0.02, L+ = 0.25, L− = 0.025) fall within the high-mass ranges, confirming that our fixed parametrization resides in a region capable of producing robust multi-task performance. Moreover, within the all-robust region, the canonical theoretical constraints of SOP are probabilistically favored: pd1 exceeds pd2 in approximately 68–76% of sampled configurations, and L+ exceeds L− in approximately 86–89% of configurations, with both constraints jointly satisfied in roughly 65–72% of cases. This probabilistic structure indicates that the subset supporting all three tasks is not merely mathematically feasible but also theoretically coherent, though not rigidly constrained to a narrow parametric regime. This alignment underscores that simultaneous success across SOR, RR, and OIP reflects genuine theoretical integration rather than an arbitrary combination of otherwise disconnected predictions.

**Table 4:** Parameter distributions within two PSP regions: All-task robust versus selective-failure region

Outcome	Parameter	Robustness criterion		
		ε = 0.05	ε = 0.10	ε = 0.15
All robust	pd1	0.080 (GSD = 2.55)	0.079 (GSD = 2.27)	0.079 (GSD = 2.11)
	pd2	0.036 (GSD = 4.56)	0.030 (GSD = 4.71)	0.028 (GSD = 3.97)
	L^+^	0.161 (GSD = 3.61)	0.198 (GSD = 3.40)	0.182 (GSD = 3.50)
	L−	0.030 (GSD = 4.68)	0.040 (GSD = 5.02)	0.028 (GSD = 4.96)
Exemplar of impaired region	pd1	0.164 (GSD = 3.23)	0.180 (GSD = 3.12)	0.178 (GSD = 3.01)
	pd2	0.009 (GSD = 3.35)	0.008 (GSD = 3.13)	0.008 (GSD = 2.98)
	L^+^	0.013 (GSD = 5.71)	0.013 (GSD = 5.68)	0.015 (GSD = 6.16)
	L−	0.078 (GSD = 5.55)	0.069 (GSD = 5.92)	0.075 (GSD = 5.81)

Entries are geometric mean of parameter within each PSP defined region; parentheses report geometric standard deviations. Regions are defined by outcome classification under each robustness threshold. “All robust” denotes parameter sets yielding robust outcomes in SOR, RR, and OIP; “Exemplar of impaired region” denotes SOR and RR robust, and OIP fail. SOR = spontaneous object recognition; RR = relative recency; OIP = object-in-place; GSD = geometric standard deviation.

A final point we wish to emphasize from the PSP concerns parameter regions that produce selective task failures, which we term “impaired SOP” configurations. As an illustrative case, we examined the subset in which SOR and RR remain robust while OIP fails. The corrected volume supporting this pattern is substantially larger than the all-robust region (21–24% vs. 3–7%, respectively), yet its probabilistic constraint structure differs markedly. In particular, whereas pd1 > pd2 remains strongly favored in the impaired region (95–97% of configurations), L+ > L− holds in only 19–22% of cases, compared with 86–89% in the all-robust region. Correspondingly, joint satisfaction of both constraints drops to approximately 18–21%, reflecting a systematic inversion of net associative influence. This inversion undermines the displacement-sensitive contrast that OIP requires, even while pd1 > pd2 is maintained to preserve the self-generated priming mechanism necessary for SOR and RR. When inhibitory associative influence dominates, the context–object association formed during preexposure is weakened or fails to adequately suppress exploration of the in-place object at test, shrinking the differential exploration between in-place and displaced objects. The existence of this dissociable failure pattern demonstrates that OIP’s mechanistic requirements are distinct from those underlying SOR and RR, and that violations of canonical constraints can yield theoretically interpretable “impaired” behavior, characteristic of a compromised configuration rather than global collapse of model performance.

While PSP identified where in parameter space predictions succeed or fail, it did not reveal why prediction magnitudes vary across those regions. To address this question, we conducted a global variance decomposition using Sobol’ sensitivity indices (Saltelli et al., 2008) to estimate the relative influence of each parameter and their interactions on variability in the predicted contrasts. To assess whether variance attribution is stable across temporal scales, we repeated the Sobol’ analysis at two delays of 2 and 8 min, which provide a minimal but theoretically meaningful perturbation of the exploration-test interval in SOP (RI for SOR and OIP; ISI for RR). For each condition, the base ordinal contrast we used was ${\widehat{A1}}_{Q} - {\widehat{A1}}_{P}$. We computed first-order indices (S_1_), which quantify the proportion of output variance attributable to each parameter independently; total-effect indices (S_T_), which capture each parameter’s total influence including all interactions; and second-order indices (S_2_), which isolate pairwise interactions. The difference between S_T_ and S_1_ indicates the extent to which a parameter’s influence depends on its alignment with other parameters. Table 5 summarizes S_1_, S_T_, and S_T_ − S_1_ by parameter and delay for each task; second-order indices for notable pairwise interactions are reported in the text.

**Table 5:** Global Sobol’ sensitivity indices for predicted contrast across tasks and delays

Task	Parameter	Delay					
		2 min			8 min		
		S_1_	S_T_	S_T_ – S_1_	S_1_	S_T_	S_T_ – S_1_
SOR	pd1	.25 [.03]	.43 [.03]	.18 [.04]	.16 [.02]	.35 [.02]	.19 [.03]
	pd2	.54 [.03]	.71 [.03]	.17 [.05]	.57 [.04]	.74 [.04]	.17 [.05]
	L^+^	.01 [.01]	.06 [.01]	.05 [.01]	.02 [.01]	.11 [.01]	.09 [.02]
	L−	.01 [.01]	.04 [.01]	.03 [.01]	.02 [.01]	.10 [.01]	.07 [.02]
RR	pd1	.41 [.04]	.81 [.05]	.39 [.06]	.41 [.04]	.82 [.04]	.41 [.06]
	pd2	.09 [.03]	.44 [.02]	.35 [.04]	.08 [.03]	.44 [.02]	.36 [.04]
	L^+^	.02 [.02]	.17 [.02]	.15 [.02]	.02 [.02]	.18 [.02]	.15 [.03]
	L−	.03 [.02]	.18 [.02]	.15 [.03]	.04 [.02]	.20 [.02]	.17 [.03]
OIP	pd1	.10 [.03]	.77 [.07]	.67 [.08]	.12 [.03]	.75 [.06]	.63 [.07]
	pd2	.05 [.03]	.50 [.05]	.45 [.06]	.04 [.02]	.40 [.04]	.36 [.04]
	L^+^	.07 [.02]	.61 [.07]	.55 [.07]	.07 [.02]	.59 [.05]	.52 [.06]
	L−	.03 [.02]	.39 [.05]	.36 [.05]	.06 [.02]	.49 [.05]	.43 [.05]

Entries report the mean variance-based Sobol’ indices of first- and total order, and their difference (interaction effect) across three independent runs of scrambled sequences; brackets report the mean 95% bootstrap CI half-width across runs. Delays were 2 and 8 min (RI for SOR and OIP; ISI for RR). SOR = spontaneous object recognition; RR = relative recency; OIP = object-in-place; S_1_ = first order; S_T_ = total-effect; S_T_-S_1_ = interaction mass.

Global variance decomposition confirmed that SOR predictions are dominated by decay parameters, rather than by learning rate parameters across both RIs. As shown in Table 5, pd2 exhibits the largest main and total effects, increasing its influence at the 8-min interval, while pd1 is the secondary contributor to predictive variance, though its contribution decreases at the 8-min interval. The contributions of L+ and L− remain comparatively small but become more influential at the longer delay. This pattern follows directly from SOP’s dynamics. The novel object is first presented at test, so all its elements are in the inactive state, yielding a maximum A1 peak that is not shaped by prior A2 occupancy. By contrast, the familiar object carries residual A2 activity. Lower pd2 retains more elements in A2, reducing their availability to transition back to A1 at test, thereby increasing the predicted contrast, whereas higher pd2 accelerates recovery from A2 to I, increasing A1 activity at test and thus reducing the contrast. As the RI increases, the A1 peak at test is progressively governed by this process, which explains why pd2’s influence strengthens at 8 min; conversely, pd1 becomes less influential as its primary role is to set the rate at which elements decay from A1 to A2, while L+ and L− gain relative importance as the stronger refractory constraint at 2-min RI diminishes, allowing associative priming to explain more of the variability in the test response. The dominant interaction across delays is pd1 × pd2 (S_2_ = .13 ± .004 at 2-min RI; S_2_ = .11 ± .005 at 8-min RI), consistent with pd1 shaping how many elements enter A2 from A1 and pd2 determining how many remain there until test. SOR leaves only a small residual variance beyond first-order and second-order terms (S_res_ = .02 ± .005 at 2-min RI; S_res_ = .05 ± .007 at 8-min RI), hence these pairwise interactions provide a comparatively faithful summary of the interaction structure.

For RR, pd1 exhibits the largest main and total effects at both delays, while pd2 provides a substantial secondary contribution through nonadditivity. This is explained because pd1 determines how many elements of each object will decay to A2 at the end of its own sample phase and, therefore, constraining A1 activation at test, whereas pd2 sets the rate at which A2 is emptied before test. The consistently large S_T_ – S_1_ effect for both pd1 and pd2 indicate that their influences are not simply additive, which is consistent with the fact that the dominant interaction across delays is pd1 × pd2 (S_2_ = .24 ± .015 at 2-min ISI; S_2_ = .24 ± .013 at 8-min ISI), reflecting that the effect of initial A2 loading depends on how much of that occupancy persists until test. Regarding L+ and L−, they remain secondary contributors, though their influence exceeds what was observed in SOR. This relatively greater contribution is consistent with the fact that RR in these simulations contrasts two familiar objects under a continuously active contextual cue, which allows associative priming to modulate both test responses. Although a longer ISI provides the remote object more time to clear A2, the variance attribution is strikingly stable across delays, implying that the same coupled mechanism governs RR across intervals with the longer ISI shifting the effect magnitude more than it reorders sensitivities. Because RR leaves a moderate residual beyond first-order and second-order terms (S_res_ = .16 ± .053 at 2-min ISI; S_res_ = .16 ± .046 at 8-min ISI), S2 terms are informative but should not be read as exhaustively accounting for all nonadditivity.

Concerning OIP, its variance structure differs sharply from both SOR and RR. As shown in Table 5, total-effect indices substantially exceed first-order indices (S_T_ >> S_1_) for all parameters across both RIs, indicating that the majority of predictive variance arises from interactions rather than additive main effects (Saltelli et al., 2008). All four parameters show large total effects, with pd1 as the dominant contributor at both delays, followed by L+. At 2-min RI, pd2 is more influential than L−, a relationship that is reversed with the longer RI. This pattern is expected because OIP introduces an explicit contextual change at test for the displaced object, so the contrast depends on how self-generated and associatively generated priming operate together. At the 2-min RI, SGP keeps A2 relatively occupied, thereby maintaining the inactive state relatively depleted, constraining the associative influence. This restriction is shaped primarily by the joint balance between pd1 and pd2. At the longer ISI, there is more time for elements to decay from A2 to I, which reduces the overall leverage of pd2 and increases the leverage of L−. Consistent with this mechanistic coupling, the largest and most interpretable interaction at both delays is pd1 × L+ (S_2_ = .14 ± .017 at 2-min RI; S_2_ = .13 ± .007 at 8-min RI), indicating that the impact of excitatory association is gated by the rate at which elements decay from A1 to A2. Importantly, second-order terms collectively explain only about one-third of total variance. The proportion of unexplained variance (S_res_ = .42 ± .009 at 2-min RI; S_res_ = .36 ± .023 at 8-min RI) suggests that much of OIP’s nonadditivity arises from higher-order interactions.

**Fig. 15:**
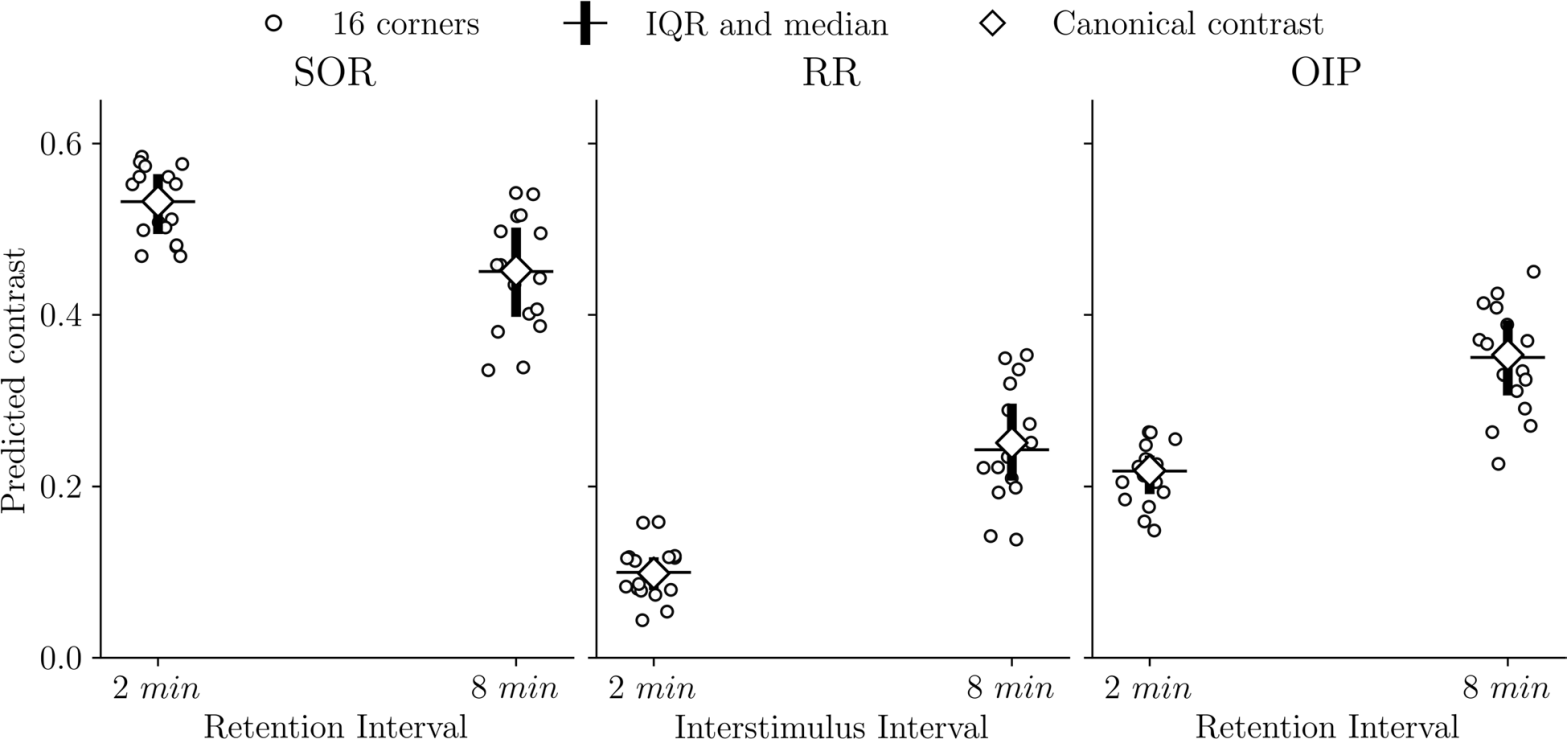
Local robustness of predicted contrast under ±10% log-perturbations in a 2 ^4 ^design across tasks and delays. Dots show the 16 corner predictions. The thin horizontal line denotes the median and the thick vertical bar the interquartile range. Diamonds mark the canonical prediction. SOR = spontaneous object recognition; RR = relative recency; OIP = object-in-place; IQR = interquartile range

Finally, following PSP, which identifies where qualitative outcomes succeed or fail across parameter space, and the Sobol’ variance decomposition, which explains which parameters dominate output variability over broad ranges, we also conducted a local, two-level full-factorial sensitivity analysis around the canonical parameter set used throughout this manuscript: pd1 = 0.1, pd2 = 0.02, L+ = 0.25, and L− = 0.025. This local analysis characterizes neighborhood robustness and directional parameter effects by evaluating the predicted contrast ${\widehat{A1}}_{Q}- {\widehat{A1}}_{P}$ at the 16 corner points of a 2^4^ design obtained via log-symmetric perturbations of ±10% for each parameter.^6^ We summarize the resulting distribution of contrast values for each task and at the same delays used in the Sobol’ analysis in Fig. 15 and report the corresponding main and two-way interaction effects from the factorial decomposition restricted to terms explaining at least 1% of local variance in Table 6.

**Table 6:** Local decomposition of predicted contrast under ±10% log-perturbations in a 2^4^ design across tasks and delays

Task	Delay					
	2 min			8 min		
	Term	Relative effect	SST	Term	Relative effect	SST
SOR	pd1	−.15	.88	pd1	−.25	.73
	L+	+.05	.10	L+	+.12	.18
	L−	−.02	.01	L−	−.08	.08
RR	pd1	+.36	.36	L−	+.38	.53
	L+	−.37	.32	L+	−.30	.34
	L−	+.35	.30	pd1	+.16	.09
				pd1 × L+	+.06	.01
OIP	pd1	−.22	.49	pd1	−.28	.62
	pd2	+.19	.39	L+	+.15	.19
	L+	+.10	.10	pd2	+.11	.10
	L−	−.03	.01	L−	−.10	.09

Effects are estimated from a two-level 2^4^ factorial design with ±10% log-symmetric perturbations around the canonical parameter set (pd1 = 0.1, pd2 = 0.02, L+ = 0.25, L− = 0.025). “Relative effect” reports each effect scaled by the canonical contrast value, quantifying how strongly that term shifts the predicted contrast relative to the canonical contrast. “SST” is the proportion of local variance attributable to each term. Only terms with SST > .01 are reported. Delays were 2 and 8 min (RI for SOR and OIP; ISI for RR). SOR = spontaneous object recognition; RR = relative recency; OIP = object-in-place; SST = sum of squares total.

As illustrated in Fig. 15, the predicted contrast values consistently matched expected outcomes across all tasks and delays. Notably, ${\widehat{A1}}_{Q} \mathrm{>} 1.15{\widehat{A1}}_{P}$ in every instance, satisfying the most stringent success threshold employed in the PSP analysis. These results indicate that the model’s qualitative predictions are locally robust to log-symmetric ±10% perturbations around the canonical parameter set. The canonical prediction (open diamond in Fig. 15) is generally centered within the local corner distribution, typically falling near the median, consistent with a well-behaved neighborhood around the canonical parameter set. At the same time, the spread across the 16 corners varies by task and delay, indicating that local sensitivity is not uniform.

Locally, the SOR contrast is governed primarily and negatively by pd1: An increase in its value shrinks the difference between the novel and familiar objects, whereas a decrease in pd1 amplifies this difference. During the test, pd1 modulates this metric through a push–pull across both objects, decreasing the novel peak while increasing the familiar peak and thereby reducing the overall discrimination. Learning rate parameters contribute to a lesser extent, although their contribution increases in the 8-minute RI. These parameters act almost exclusively on the familiar object, where L+ reduces the peak and thus increases the difference, whereas L− increases the peak and thus attenuates it, consistent with excitatory and inhibitory associative processes.

In RR, the same three main effects as in SOR explain the local variance of the contrast but in the opposite direction and comparable contributions at 2-min ISI. Because both objects are familiar, their peaks increase with higher pd1 and L−, and decrease with higher L+, such that the discrimination inherits these directions. At the longer RI, however, L+ and L− increase their contribution, consistent with the remote object having more time for its net associative strength to weaken. RR is also the only task in which an interaction exceeds the reporting threshold. This effect between pd1 and L+, although modest, indicates that excitatory learning depends slightly on the activation of A2 via pd1.

Finally, in OIP, as in SOR and RR for the familiar and the recent objects, respectively, the in-place item is governed primarily by pd1, L+, and L−. By contrast, the displaced object is governed mainly by pd2. At local level, under contextual mismatch conditions, the AGP for the displaced object is less effective than that for the in-place object, such that its peak value is determined more directly by A2 persistence. Accordingly, increasing pd2 accelerates the emptying of A2, thereby increasing the pool of available elements at test and, consequently, the displaced object peak.

Taken together, these local results complement the global Sobol’ decomposition. The latter quantifies variance attribution across broad parameter ranges, without any a priori theoretical constraint, and thus may traverse qualitatively distinct dynamics, whereas the former characterizes directional effects and near-additivity within a bounded neighborhood around the canonical parameter set. This distinction is particularly relevant for parameters such as pd2. For instance, in SOR, pd2 contributes negligibly within the ±10% neighborhood because the contrast is mediated primarily by pd1 and learning rate parameters. However, across wider ranges, pd2 becomes influential by shifting A2 occupancy enough to reweight priming processes and alter the leverage of other parameters.

The global and local analyses we conducted were performed on simplified theoretical instantiations of the target processes. The empirical experiments we simulate involve substantially richer structure, including multiple objects and contextual nodes, counterbalancing schemes, and several implementation considerations, such that the present results should be interpreted as regime-specific diagnostic rather than exhaustive sensitivity rankings for every experimental detail. Nevertheless, this analysis remains highly informative: it maps where the core mechanisms succeed or fail, identifies which parameters can dominate variability over broad ranges, and clarifies the directional, near-additive processes operating in the neighborhood around the parameter set used throughout the manuscript.

## Discussion

Our primary aim was to deliver a quantitative account of how the SOP model captures core regularities in three object recognition procedures. Without relying on parameter tuning, most simulations reproduced the expected ordinal patterns across SOR, RR, and OIP tasks, consistent with benchmark datasets and with the logic of self-generated and associatively generated priming. These results suggest that the SOP framework provides a parsimonious and mechanistic account of recognition memory. We also provide a detailed quantitative adaptation of the SOP model to these procedures, together with Python code for further assessment.

The SOP framework also yields falsifiable predictions in this domain. In object-recognition tasks, we assume two sources of priming: associative priming (context to object) and self-priming (residual activity of the object’s own representation). In SOP terms, associative priming arises when the test context promotes the object’s elements from I to A2; self-priming arises when recently presented object elements remain in A2 at test, making reentry into A1 less likely and thus reducing exploration. Because these mechanisms depend on different computations in SOP, each should exhibit its own generalization gradient as similarity varies between the familiar item and the test item: one gradient reflecting how context-to-object associations generalize, and a second gradient reflecting how much of the familiar item’s A2 state is shared with the similar test item. Longer delays should primarily thin self-priming (faster decay of A2); switching to a different context should primarily reduce associative priming. Mapping the shape and slope of both gradients while crossing context (same vs. different) and delay provides a direct diagnostic of which source of priming is dominant under which boundary conditions.

These considerations also predict informative interactions in relative-recency designs where two objects are sampled at different times in the same context. If generalized self-priming from the first to the second object is substantial because the objects are similar, the usual “remote greater than recent” advantage should shrink as similarity increases. Conversely, when objects are dissimilar but context is held constant, associative priming can still support both items at test and partially offset time-based differences. By crossing object similarity (discrete levels), the sample-to-sample interval (short to long), and context (same vs. different), one can determine whether relative-recency effects arise chiefly from temporal separation in self-priming, from shared associative support, or from their interaction.

Furthermore, because most recognition studies use within-subjects designs (efficient but susceptible to cross-trial generalization between cues and contexts) it is advisable to replicate key tests with between-subjects variants (unique familiar/test pairs and/or contexts per participant). Converging evidence across within- and between-subjects designs will render the estimated gradients, and their task-specific consequences, more general and less dependent on design-induced carryover. However, between-subjects designs are less efficient: they absorb more variability across participants and items, so they typically require larger sample sizes to achieve comparable precision. When feasible, analyses should use multilevel mixed-effects models that include random effects to account for individual (and item) variability. We therefore view between-subjects designs as confirmatory complements to within-subjects tests, not replacements.

A second line of work targets the predicted nonlinearity of self-priming as a function of the interval between two presentations of the same item. In SOP, the decrement at the second presentation depends on how many elements remain in A2 from the first presentation and have not yet returned to inactivity, making them unavailable for reactivation by the second input. However, at very short ISIs, by the time the second presentation occurs, very few elements from the first presentation have decayed into A2. Instead, many still remain in A1, so when the second input arrives it summates with that residual A1 activity, producing relatively little suppression and potentially even an increase in responding to the second stimulus. At intermediate ISIs, more elements have accumulated in A2 while relatively few have yet returned to inactivity, so suppression at the second presentation is maximal. At long ISIs, the effect of the first presentation on the second becomes minimal because activity in both A1 and A2 has largely dissipated. In RR, the self-priming account therefore predicts that the remote-over-recent advantage should be maximal at intermediate intervals. The net prediction is, therefore, an inverted-U relation between the ISI and decrement at the second presentation, with the greatest suppression at intermediate intervals. To test this prediction experimentally, one could manipulate ISI within subjects using a broad, log-spaced grid (from seconds to tens of minutes), and replicate or counterbalance across subjects to limit cross-trial carryover.

A third avenue for future work concerns associative priming in the OIP test. In SOP, the retention of associative support reflects a context-object link formed at the time of sampling; critically, that link is susceptible to extinction when animals remain in the training context without the objects. Extinction should therefore increase with the amount of in-context exposure during the RI, shrinking the displacement advantage at test. By contrast, if the RI unfolds outside the training context, SOP predicts little or no extinction of the context-object association (the displacement advantage should remain largely invariant across delays, aside from any global change in exploration due to decay of self-priming).

A straightforward experiment is a 2 × K design crossing retention context (in-arena vs. out-of-arena) with interval length (K levels spanning minutes to hours). Within each level, equate handling and arousal (e.g., yoked exposure in a neutral holding cage), preexpose the arena to reduce context novelty, and index extinction as the difference in exploration of displaced versus in-place objects at test. SOP predicts a monotonic decline in this OIP difference with longer in-arena intervals (an extinction curve), but a flat function across out-of-arena intervals. To confirm that the effect is associative rather than self-priming, include controls that weaken context-object links without changing timing (latent inhibition, blocking, brief context-only sessions) and verify that these selectively trim OIP while leaving spontaneous recognition and RR comparatively intact. Replicating the key contrast in a between-subjects variant (unique contexts/pairs per participant) will guard against cross-trial generalization and strengthen the inference.

A further avenue for future work concerns the mechanisms that can contribute to RR. As noted briefly in our schematic description of Fig. 1, RR is often explained primarily in terms of temporal proximity: the recent object is more strongly self-primed at test than the remote object, which reduces its A1 and exploration, yielding a remote-over-recent preference. At the same time, SOP also permits associative contributions that can complement, and in some circumstances potentially substitute for, a predominantly self-priming account.

One associative route is extinction-like updating of context–object support across samples when both samples occur in the same physical context. In the second sample, the context can retrieve the remote object into A2 even when it is absent, and, concurrently, context A1 with object A2 accrues inhibitory learning, weakening the net context–remote association. This reduces contextual priming of the remote object at test and can therefore amplify the remote-over-recent preference. This context-based mechanism was implemented in the simulations reported above.

A second associative route, omitted here for simplicity, is item-based learning between the objects. In SOP, inhibitory links can form when one representation is active in A1 while another is active in A2 (Sanderson, 2016). Applied to RR, if remote elements are active in A2 during sampling of the recent object, the recent object could acquire inhibitory control over activation of the remote object at test. This item-based inhibition is likely less parsimonious for standard RR findings at short intervals, but it becomes a plausible extension in procedures that report robust order effects after long delays, where residual self-priming is expected to be minimal (Mitchell & Laiacona, 1998). Moreover, because remote A2 during the second sample can be supported by context-mediated retrieval rather than by lingering self-priming alone, the opportunity for item-based inhibitory learning need not be tightly coupled to the sample-to-sample interval and may instead be more sensitive to contextual similarity and the strength of context–object associations.

Together, these mechanisms suggest that RR can reflect a mixture of temporal and associative influences, with self-priming plausibly dominating in many canonical procedures, whereas associative routes become increasingly important in long-interval variants and multi-item temporal order designs. In this regard, Barker (2019) reported robust serial order discrimination that was largely insensitive to manipulating the interval across a wide range. Within SOP, such delay-insensitive order effects are naturally more compatible with associative mechanisms than with a purely time-dependent self-priming account. In particular, extinction-like updating of context–object support across successive samples and, if implemented, item-based inhibitory learning could both contribute to stable order gradients even when residual self-priming is minimal.

At the same time, Barker et al. (2019) also reported a pattern that complicates a straightforward associative interpretation (although see Nitka et al., 2020, for commentary on this article). In one analysis, each serially presented object was tested separately against a novel object, and all familiar objects showed similar novelty deficits regardless of their serial position. If within-list order discrimination were driven by systematic differences in net associative support across list items, one would typically expect corresponding graded differences in each item’s discrimination relative to a novel comparator. The apparent dissociation between a robust within-list order effect and the absence of graded novelty discrimination therefore places an informative constraint on SOP-based associative accounts, and it motivates targeted future simulations and experiments that jointly model multi-item sampling, context structure, and test format, as well as further replication of this specific pattern across procedures and laboratories.

Another theoretical aspect of SOP, not treated above, concerns how the model accommodates dishabituation-like effects and their manifestations as interference in object recognition. In classic habituation, dishabituation is the transient recovery of a habituated response when an innocuous “dishabituator” is interposed between two presentations of the target (Whitlow, 1975). Humphrey (1933) anticipated that habituation is fragile, reversible, and sensitive to global changes in stimulation conditions. By analogy, in object recognition, an interposed distractor can reduce novelty preference. For instance, in relative-recency sequences, inserting a salient event between sampling and test can make the familiar item appear “newer,” thereby shrinking the discrimination. Conversely, inserting a salient event soon after the first sample would make this remote event appear “newer” and enhance the size of the recency effect (Keep & Bonardi, in preparation).

SOP explains this with Wagner’s (1981) distractor rule, which formalizes a limited-capacity constraint on stimulus processing: at any moment, there is an upper bound on the total A1 and A2 activity that can be supported across concurrently processed stimuli. In the model, this bound is implemented by increasing the decay parameters pd1 and pd2 in proportion to the summed A1 + A2 activity generated by all stimuli present. If a distractor is processed while the target trace is still active, the target’s elements return more quickly from A1 and A2 to the inactive pool. This “clearing” releases the target from self-priming, so that at test, more of its elements are available to enter A1, expressed behaviorally as increased exploration of the familiar object and thus poorer recognition (Uribe-Bahamonde et al., 2021; Wagner, 1981). In habituation terms, the distractor produces a recovery; in recognition terms, it produces interference.

This distractor mechanism is consistent with reports that additional object exposure impairs SOR when it occurs either before preexposure or during the RI within a critical time window (proactive and retroactive interference, respectively; Martínez et al., 2014; Villar et al., 2017; Winters et al., 2007). Notably, retroactive interference typically induces greater impairment than proactive interference, plausibly because the interfering exposure is temporally closer to the test or because processing during preexposure increases memory load (Qiao et al., 2023).

SOP accommodates both patterns: interference scales with the amount and timing of concurrent activity that overlaps the target’s A1→A2→I trajectory, predicting more substantial effects for distractors that fall nearer the period of maximal A2 occupancy and for conditions that elevate overall processing demands. This alignment between the distractor rule and the proactive/retroactive dissociation suggests a valuable bridge between the literatures on dishabituation and recognition memory, and it points to targeted manipulations of distractor load, similarity, timing, and context as decisive tests in future work.

Arousal can also modulate preferential exploration. In SOR, acute stress induced by 4-h restraint before the preexposure phase increased exploration of the familiar object at a 1-h RI, an effect absent at 24 h—consistent with a transient emotional state that promotes novelty avoidance rather than a durable memory deficit (Vargas-López et al., 2015). Conversely, when rats spend the RI in a familiar, quiet, dark box, performance on the object-location task (a spatial SOR variant) is better than when animals return to home cages with cage-mates, implying that reduced sensory stimulation during the RI benefits long-term retention (Arkell, 2021).

Wagner and Brandon’s (1989) extension of SOP offers a principled account: a stimulus engages parallel sensory–motor and emotive–arousing A1/A2 sets, and arousal can elevate the intensity parameter p1 for other stimuli, making response efficacy state-dependent (Jorquera et al., 2024). In object-recognition procedures, an acute stressor encountered during sampling, retention, or test would transiently raise p1 for any object—including those already explored—thereby boosting A1 entry at test for both items and compressing their exploration difference, impairing discrimination. In parallel, higher arousal typically accompanies greater overall stimulation, which in SOP increases total A1+A2 activity and thus the effective decay rates pd1 and pd2; this accelerates the clearance of the target trace and further erodes its mnemonic advantage. Quiet RIs, by contrast, should lower arousal and sensory load, yield the opposite pattern, and preserve performance.

Our SOP analysis offers a different perspective on recognition memory to the single- and dual-process models referred to in the introduction (Berry et al., 2012; Wixted, 2007; Yonelinas, 2002). However, SOP should probably be treated more as an elaboration of these traditional accounts than as an alternative, and one that cuts across the traditional conceptualizations. Traditional single-process and dual-process accounts are designed to explain both *familiarity*—the sense of having encountered an item before—and *recollection*—using the item to retrieve information about the encoding experience (Aggleton & Brown, 2006; Mandler, 1980; Yonelinas, 2002). Our analysis has focused solely on explaining the *familiarity* component of recognition; we have not dealt with recollection at all in this article. However, recollection falls out very simply from our analysis: We would simply examine the degree to which presentation of the target item at test can retrieve elements of, for example, the context in which it occurred in the sample phase. Typically animal experiments using the SOR procedure do not examine recollection in this way, but it would be very easy to do. For example, animals could be exposed to object P in context x, and object Q in context y, and then tested in a context comprising elements of both x and y. At test object P should, by selectively associatively priming context x, reduce exploration of context x relative to context y, while object Q would have the opposite effect. This would be a behavioral indicator that the recognized item can access information about the training context. Moreover, unlike familiarity, SOP asserts that recollection relies solely on associatively-generated priming.

Our account asserts that SOP explains recognition in terms of the associative, AGP, and nonassociative, SGP, priming processes. In this sense it is unambiguously a dual-process model—but these two processes do not map simply onto familiarity and recollection in the same way as in their human counterparts. According to SOP, *both* familiarity and recollection can show dependence on associative priming, AGP, whereas only familiarity can be influenced by self-generated priming, SGP. Thus it is not a dual- process account in the traditional sense, as these two priming processes do not map onto familiarity and recollection in a one-to-one manner. Thus SOP’s conceptualization of recognition memory phenomena is related to, yet different from, that offered by existing accounts.

More broadly, SOP offers a rich set of mechanisms that can be informative in their own right, but comprehensive simulations that combine multiple extensions quickly become high-dimensional and difficult to constrain. Increasing mechanistic breadth can also increase the risk of overfitting, so we have prioritized parsimonious implementations that target a core, replicable set of recognition findings while treating less common but theoretically informative effects as opportunities for focused future work. At the same time, an important source of constraint on these extensions can come from neurobiological evidence, because neural interventions and circuit-level analyses can help identify which components of the model are most plausibly altered by a given manipulation.

With this scope in mind, it is pertinent to acknowledge a complementary frontier that links behavior to neural analyses. Most object recognition studies deploy lesions, pharmacological manipulations, or transgenic models, whereas our simulations have focused on control animals. These interventions have targeted multiple systems, including excitotoxic and neurotoxic lesions of hippocampal and cortical regions in rats (Albasser et al., 2009; Good et al., 2007; Jones et al., 2012; Mitchell & Laiacona, 1998; Mumby et al., 2007; Norman & Eacott, 2005; Tam et al., 2014); surgical disconnections such as the fornix (Mogensen et al., 2004; Norman & Eacott, 2004, 2005; Simpson et al., 1998), the mammillothalamic tract (Nelson & Vann, 2014, 2017), and perforant path transections (Cooper et al., 2022) in rats; and transgenic mouse models associated with Alzheimer’s disease (Bonardi et al., 2016, 2021) or schizophrenia (Landreth et al., 2021). These approaches aim to dissociate components of recognition memory and to chart the contributions of different brain regions to object, temporal, and spatial processing.

More generally, neurobiological constraints can also help reduce theoretical underdetermination when extending computational models, by narrowing which parameter changes are plausible for a given manipulation (Soto, 2019). Although SOP is not a neural circuit model, it has motivated biologically informed interpretations and circuit-level analogies in other learning domains. For example, Hawkins and Kandel (1984) noted parallels between interacting facilitatory and inhibitory processes in simple learning circuits and the reciprocal influences often attributed to SOP’s activity states. Likewise, Wagner and Donegan (1989) discussed a mapping of SOP processing onto cerebellar circuitry in rabbit eyeblink conditioning. Such structural correspondences are best viewed as inspirational rather than confirmatory, but they illustrate how neural evidence can guide which aspects of a process model deserve emphasis and how intervention effects might be translated into constrained parameter perturbations.

In the recognition-memory literature, a common neurocognitive distinction is between cortical systems that support object representations and familiarity and hippocampal systems that support contextual and relational retrieval (Aggleton & Brown, 1999; Brown & Aggleton, 2001; Eichenbaum et al., 2007). Within a cautious functional interpretation of SOP, self-generated priming can be viewed as arising from the persistence and decay of object representations that are plausibly expressed in perirhinal and related cortical networks, whereas retrieval-generated priming depends on learned context-to-object links and contextual cueing, which is more plausibly supported by hippocampal and parahippocampal circuitry. This mapping is not intended as a one-to-one identification of SOP states with specific neural structures. Rather, it provides a neurobiologically informed constraint that can guide which parameter changes are plausible under particular interventions and which procedures should be most sensitive to those changes.

Sanderson (2016) provides a detailed analysis of how SOP can be used to interpret such findings, with particular emphasis on hippocampal involvement and on the competitive interaction between self-generated and retrieval-generated priming. Importantly, several themes in that analysis connect directly to the associative routes discussed above for RR and OIP. In RR, we noted that a common physical context can support extinction-like updating of context-to-object links across samples, and we also noted that item-based inhibitory learning is a plausible SOP-consistent extension for long-interval order effects (Sanderson, 2016). In OIP, we emphasized that retention of contextual support should be sensitive to in-context exposure during the RI. If hippocampal and related circuitry are especially important for associative retrieval and the expression of context-dependent support, then hippocampal manipulations should disproportionately affect these context-dependent components. This provides a principled way to connect apparent neural dissociations back to model mechanisms rather than treating tasks as categorically distinct.

A particularly informative case highlighted by Sanderson (2016) concerns hippocampal involvement in standard object recognition, where some studies report little or no impairment whereas others report reliable deficits. SOP naturally suggests a quantitative interpretation in which standard recognition reflects the joint influence of self-generated priming and context-generated priming. If a manipulation primarily disrupts associative learning or associative retrieval, then impairment on the standard task can be modest under conditions in which self-generated priming predominates but should become more detectable as procedures rely more on associative contributions. This procedure-sensitivity account, emphasized by Sanderson (2016), yields specific predictions. Context-dependent variants should show larger and more reliable impairments than standard recognition when associative mechanisms are weakened. Standard recognition should be most vulnerable under conditions that attenuate recency-dependent priming, such as longer RIs, weaker sampling exposure, or test conditions that reduce recency-driven suppression at test, because performance must then rely more heavily on retrieval-generated priming. Conversely, when tests occur at short delays under strong recency conditions, standard recognition may appear spared even if associative mechanisms are compromised, because self-generated priming remains sufficient to support novelty preference (Bonardi et al., 2016, 2021).

These considerations motivate an impaired SOP approach in which manipulations are mapped onto specific parameters and their predicted signatures are compared across tasks. For example, a selective associative impairment can be formalized as a reduction in excitatory learning relative to inhibitory learning, a reduction in the effective retrieval drive from contexts to objects that governs promotion into A2, or reduced effective activation of context nodes at test. By contrast, manipulations that alter the time course of priming can be formalized as changes in pd1 or pd2. Altering pd2 changes how quickly elements return from A2 to the inactive pool and therefore directly changes the persistence of self-generated priming across delays. Altering pd1 changes how quickly elements leave A1 for A2, which can change the balance between immediate responding and the accrual of associative strength. Implementing such parameter changes in quantitative simulations would enable explicit predictions about when standard recognition should be spared versus impaired, when context-dependent deficits should dominate, and how delay functions should shift under different intervention profiles (Sanderson, 2016).

A distinctive advantage of SOP in this domain is that the two sources of priming interact competitively, so impairing one process can, under some conditions, enhance another process. In SOP, strong recency-dependent priming can limit associative learning and later retrieval by constraining A1 availability and reducing the opportunity for A1 co-occurrence that supports excitatory learning. Consequently, interventions that weaken self-generated priming can sometimes release capacity for A1 activation and strengthen the associative contribution, which implies that a manipulation can produce opposite behavioral effects at different delays. A clear illustration is the GluA1 work discussed by Sanderson and colleagues, in which altering activation dynamics can yield impaired performance at short intervals but enhanced performance at long intervals, consistent with enhanced associative learning alongside reduced recency-dependent expression (Sanderson, 2016; Sanderson et al., 2009). These delay-dependent reversals are difficult to reconcile with accounts that treat recognition as a single monotonic memory-strength process, but they follow naturally from SOP’s competitive architecture and provide strong leverage for testing impaired SOP variants.

Taken together, the present work advances SOP from a retrospective fit to a generative framework. We have identified concrete behavioral signatures for future tests under intact processing, including similarity-based generalization for self-generated and context-generated priming, the inverted-U prediction for self-generated priming over interval, and context-dependent retention in OIP. We have also extended the account to interference and arousal through the distractor rule, and we have outlined how neural and genetic manipulations can be incorporated as principled parameter changes. The next step is to pair behavioral experiments with model-based inference that estimates the relevant parameters and tests their predicted boundary conditions across intact and impaired preparations. By aligning task design, quantitative predictions, and neural intervention within a single formalism, SOP offers a heuristic path toward more decisive research on recognition memory.

## Transparency and openness

To promote reproducibility and transparency, all simulation scripts used in this paper are openly available on the Open Science Framework (OSF; https://osf.io/8x7vn/files) (Galarce et al., 2025).

## Data Availability

Not applicable.

## Footnotes

1 While most studies involve brain manipulations such as targeted lesions, we focused exclusively on control group data to establish a baseline model of object recognition. Future work could extend our simulations using SOP to incorporate data from lesioned subjects and analyze the resulting behavioral differences. However, this falls outside the scope of the present article.

2 Strictly speaking, the contexts present in the arena share many common elements; however, we assumed that the contexts are orthogonal because it is the unique elements of each context that mediate the effects of interest.

3 While Tam et al. (2014) did not report exploration times for each object separately, they provided the discrimination ratio, calculated as (x1 − x2)/(x1 + x2), and total exploration time x1 + x2, where x1 represents the novel/remote/displaced object and x2 the familiar/recent/undisplaced object. Individual exploration time for each object can be derived from these reported values.

4 Tam et al. (2014) did not report *SEM* for individual objects. We derived these values from the reported discrimination ratio *SEM*, assuming zero covariance between objects for simplicity.

5 In the RR + OIP task of Good et al. (2007), we computed three contrasts to isolate its components: RR (remote in-place minus recent in-place), OIP (recent displaced minus recent in-place), and RR + OIP (remote displaced minus recent in-place). Because these contrasts are derived from the same procedure, its total weight was split equally across the three contrasts.

6 The ±10% perturbation magnitude was selected as the largest log-symmetric neighborhood around the canonical parameter set in which the contrast ${\widehat{A1}}_{Q}-{\widehat{ A1}}_{P}$ remained well approximated by a low-order local expansion (i.e., main effects and two-way interactions) across all tasks and delays, as evidenced by minimal curvature and negligible higher order remainder, consistent with standard practice for 2^k^ factorial designs (Montgomery, 2013).

## Code availability

To promote reproducibility and transparency, all simulation scripts used in this paper are openly available on the Open Science Framework (OSF) at https://osf.io/8x7vn/overview?view_only=5afe6a7a0d1744669e9c06eb1a5e59b1
